# Relative effectiveness of medications for opioid-related disorders: A systematic review and network meta-analysis of randomized controlled trials

**DOI:** 10.1371/journal.pone.0266142

**Published:** 2022-03-31

**Authors:** Jihoon Lim, Imen Farhat, Antonios Douros, Dimitra Panagiotoglou

**Affiliations:** 1 Department of Epidemiology, Biostatistics, and Occupational Health, McGill University, Montreal, QC, Canada; 2 Department of Medicine, McGill University, Montreal, QC, Canada; 3 Centre for Clinical Epidemiology, Lady Davis Institute, Jewish General Hospital, Montreal, QC, Canada; 4 Institute of Clinical Pharmacology and Toxicology, Charité—Universitätsmedizin Berlin, Berlin, Germany; University of Phayao, THAILAND

## Abstract

**Introduction:**

Several pharmacotherapeutic interventions are available for maintenance treatment for opioid-related disorders. However, previous meta-analyses have been limited to pairwise comparisons of these interventions, and their efficacy relative to all others remains unclear. Our objective was to unify findings from different healthcare practices and generate evidence to strengthen clinical treatment protocols for the most widely prescribed medications for opioid-use disorders.

**Methods:**

We searched Medline, EMBASE, PsycINFO, CENTRAL, and ClinicalTrials.gov for all relevant randomized controlled trials (RCT) from database inception to February 12, 2022. Primary outcome was treatment retention, and secondary outcome was opioid use measured by urinalysis. We calculated risk ratios (RR) and 95% credible interval (CrI) using Bayesian network meta-analysis (NMA) for available evidence. We assessed the credibility of the NMA using the Confidence in Network Meta-Analysis tool.

**Results:**

Seventy-nine RCTs met the inclusion criteria. Due to heterogeneity in measuring opioid use and reporting format between studies, we conducted NMA only for treatment retention. Methadone was the highest ranked intervention (Surface Under the Cumulative Ranking [SUCRA] = 0.901) in the network with control being the lowest (SUCRA = 0.000). Methadone was superior to buprenorphine for treatment retention (RR = 1.22; 95% CrI = 1.06–1.40) and buprenorphine superior to naltrexone (RR = 1.39; 95% CrI = 1.10–1.80). However, due to a limited number of high-quality trials, confidence in the network estimates of other treatment pairs involving naltrexone and slow-release oral morphine (SROM) remains low.

**Conclusion:**

All treatments had higher retention than the non-pharmacotherapeutic control group. However, additional high-quality RCTs are needed to estimate more accurately the extent of efficacy of naltrexone and SROM relative to other medications. For pharmacotherapies with established efficacy profiles, assessment of their long-term comparative effectiveness may be warranted.

**Trial Registration:**

This systematic review has been registered with PROSPERO (https://www.crd.york.ac.uk/prospero) (identifier CRD42021256212).

## Introduction

Opioid use disorder (OUD), including opioid dependence and addiction, is a problematic opioid use commonly characterized by tolerance and withdrawal symptoms. Adverse health outcomes associated with OUD include overdose, infectious diseases (e.g., AIDS; hepatitis C; and skin, soft tissue, and vascular infections), suicide, and death in severe cases [1–5]. A recent study on OUD burden revealed a global estimate of 40.5 million people who suffer from opioid dependence and 109,500 deaths from opioid overdose (including both accidental and intentional cases) in 2017 [6]. In response to the severity of OUD disease burden, several treatment pathways have emerged, with opioid maintenance therapy (OMT) as the most effective strategy.

OMT involves the use of opioid substitutes (e.g., buprenorphine, methadone, or naltrexone) to treat and manage addiction to opioids such as heroin, fentanyl, hydromorphone, and oxycodone [7]. Compared to other treatment pathways (e.g., detoxification, residential services, and behavioural interventions) or no treatment, OMT has lower risk of overdose hospitalizations [8], mortality [9], and frequency of injection drug use and needle sharing [10–12]. With mounting evidence of its effectiveness, OMT has undoubtedly become the gold standard for treating opioid addiction and dependence, and public health stakeholders around the world have sought to expand access to medications for OUD in order to reduce the individual- and population-level burdens of OUD [13–15].

Researchers from the United States, Canada, and European nations (e.g., the UK, France, Germany, Spain, and Finland) have adopted guidelines or reached a consensus to prescribe buprenorphine and methadone as first-line OMT treatments against OUD [15–18]. At the same time, there have been growing interests in understanding the efficacy of other medications for OUD, such as naltrexone [19, 20] and slow-release oral morphine (SROM) [21–23]. For example, recent clinical practice guidelines from Canada and the United States have outlined expectations of professional conduct in relation to prescribing these medications in addition to buprenorphine and methadone [17, 18]. These OMT guidelines reflect advances in clinical practice, and the availability of a wider range of therapeutic options for OMT medications may reduce OUD burden [14]. However, no review to date has compared treatment efficacy between several different OMT medications. Despite the availability of multiple medications, previous meta-analyses have been limited to pairwise comparisons of buprenorphine vs. methadone [24], SROM vs. methadone [21], or OMT medications vs. no maintenance treatment [20, 25]. In the absence of randomized controlled trials (RCT) comparing other combinations of treatment pairs, especially those involving naltrexone or SROM, their treatment efficacy relative to that of buprenorphine or methadone remains poorly understood. We undertook a systematic review with a network meta-analysis to establish which medications out of buprenorphine, methadone, naltrexone, and SROM have superior efficacy profile. This study unifies findings from different healthcare practices around the world and generates evidence to strengthen clinical treatment protocols for the most widely prescribed OMT medications.

## Materials and methods

We conducted this systematic review in accordance with a pre-specified protocol (PROSPERO number: CRD42021256212), and we reported our findings following the Preferred Reporting Items for Systematic Reviews and Meta-Analyses for Network Meta-Analyses (PRISMA-NMA) (**S1 Table**) [26].

### Search strategy

We conducted a systematic literature search of studies comparing medications for OUD among people with opioid-related disorders in MEDLINE, EMBASE, PsycINFO, and Cochrane CENTRAL databases. We also scanned the bibliographies of the included articles and searched through the first 10 pages of Google Scholar (search terms: ‘randomized controlled trials opioid use disorder’) to identify additional references in the grey literature. We tailored our search strategy to each database, and search terms and keywords included those related to buprenorphine, methadone, naltrexone, slow-release oral morphine, and opioid-related disorders (see **S2 Table** for Search Strategy). There were no restrictions on the language of publication. Initial search was conducted on June 7, 2021, and it was updated on February 12, 2022.

### Inclusion and exclusion criteria

#### Population

This study included adult patients with problematic opioid or heroin use who were receiving pharmacotherapy, including buprenorphine, methadone, naltrexone, and SROM. Problematic use included OUD, opioid dependence, and opioid addiction as well as conditions specific to the use of heroin. These conditions were ascertained by the Diagnostic and Statistical Manual of Mental Disorders (DSM-3, -4, or -5) or the International Classification of Diseases (ICD) criteria. We excluded women who were pregnant in order to mitigate heterogeneity in study population that could arise from scientific and ethical complexities involving these patient population groups.

#### Interventions and comparators

The main intervention for this study was pharmacotherapy for OUD including buprenorphine, methadone, naltrexone, and slow-release oral morphine. We included studies that compared any of the described intervention medications with each other or with a control group (e.g., placebo, standard of care, or no treatment). For inclusion, these interventions needed to be administered to treat OUD. Studies with one of the four intervention medications in one arm but another medication that is not one of the four intervention medications or controls (e.g., heroin or clonidine) were excluded. This was because they were not described in the latest best practice guidelines and consensuses for opioid maintenance treatment, and therefore did not reflect the most up-to-date clinical practice around the world (e.g., North America and Europe). In addition, we excluded from our analyses RCTs that compared different treatment regimens of the same medication, including studies where only the dosage, setting, or the route of administration (e.g., sublingual vs. injectable) differed between treatment groups.

For the multi-arm trials, we took the following analytic approach to evidence synthesis. First, in studies with three or more treatment arms, we extracted all intervention information but included only the eligible arms for evidence synthesis, following the approach taken by Rice et al. 2020 [27]. Second, in studies with two or more treatment arms with the same intervention (e.g., two arms with buprenorphine and two arms with methadone), we categorized each arm as ‘low dose’, ‘moderate dose’, or ‘high dose’ and treated two intervention arms with different medications that have the same categorization as a separate study. For example, if the four intervention arms were low dose buprenorphine, low dose methadone, moderate dose buprenorphine, and moderate dose methadone, the two low dose arms were included in the analysis as one study and the two moderate dose arms as another study. Third, in other trials with a small sample size in each arm, we combined multiple treatment arms with the same intervention into a larger single arm, if applicable, as had been done in earlier meta-analyses [20, 24, 25].

#### Outcomes

The primary outcome of interest was treatment retention at the end of the study. We defined treatment retention for each intervention or control arm as the number of participants who completed or remained in the study (did not withdraw) divided by the total number of participants who were randomized to a specific treatment in the beginning of the study. The secondary outcome of interest was opioid use based on urinalysis. Opioid use could be reported as either abstinence from opioids or illicit opioid use (e.g., heroin), and the definition of abstinence could vary by study duration or the number of times of opioid use. Therefore, for each intervention or control arm, we defined opioid use in two different ways: (1) Percentage of urine samples that were positive for opiates at the end of the study, and (2) Number of patients who had at least one urine sample that was positive for opiates at the end of the study out of all those randomized to each arm in the beginning of the study.

#### Study design

We included RCTs that compared any of the four medications for OUD, namely buprenorphine, methadone, naltrexone, and SROM against each other for any treatment regimen (dose, frequency, timing, duration, and route of administration), placebo, standard of care, or no treatment. We restricted our study design to RCTs because they were best suited to address the relative efficacy of the pharmacotherapies on retention and opioid use, while eliminating confounding present in other study designs. Consequently, observational studies, case series, and case reports were excluded from our analysis. To identify additional eligible studies, we inspected earlier systematic reviews and meta-analyses, but these systematic reviews and meta-analyses themselves were not included in the qualitative and quantitative evidence syntheses.

### Screening

Two independent reviewers (JL and IF) screened titles and abstracts, and then conducted a full-text review of all articles retrieved from the databases for eligibility (study selection) based on specified inclusion/exclusion criteria. We conducted title and abstract screening using a liberal accelerated approach, in which only one reviewer was needed to include a citation, while two reviewers were needed to exclude a citation [28]. At this stage, articles that were judged to be potentially relevant underwent a full-text review, which two reviewers performed independently and in duplicate. Disagreements between the two reviewers were resolved through consensus or adjudication by a third independent reviewer (DP), if necessary.

### Data extraction

Two reviewers (JL and IF) conducted data extraction using a pre-piloted, standardized data extraction form (**S3 Table**). Data were independently extracted by a single reviewer (JL) and were verified by a second reviewer (IF). Disagreements were resolved through consensus or adjudication by a third independent reviewer (DP), if necessary. Extracted information included publication traits (year, setting/country, and funding source), study characteristics (trial design, trial duration, intervention type, comparator type, treatment regimen for each arm, number of participants randomized [total and for each arm], inclusion/exclusion criteria), basic participant characteristics (age and sex), and outcome data (number of participants retained and results from urinalysis for detecting opiates, including morphine and heroin). If multiple studies reported on the same cohort, we included only the most recent article with the most up-to-date study information.

### Risk of bias assessment

Two reviewers (JL and IF) independently assessed the risk of bias for all included studies. We used Version 2 of the Cochrane Risk of Bias (RoB 2) tool to examine potential biases in five domains [29]. These include biases in randomization process, deviations from intended intervention, missing outcome data, measurement of the outcome, and selection of the reported results. Based on the assessment of each domain, we determined the overall risk of bias as one of ‘low risk of bias’, ‘some concerns’, and ‘high risk of bias’, where the overall risk was determined by the highest risk assigned in any individual domain.

### Review of network geometry

We constructed network graphs to visualize the overall structure of the comparisons in our network. The nodes of the network graph represent the competing treatments, and an edge connects them if at least one RCT compared these corresponding treatments. We examined which treatments were compared directly (head-to-head comparisons) or indirectly (through one or more common comparators) and the amount of evidence generated from each comparison.

### Strategies for evidence synthesis

We conducted a network meta-analysis of available direct and indirect evidence using a Bayesian framework to account for the correlation between treatment effects by different comparisons in multi-arm trials [30]. We chose to conduct network meta-analysis using the Bayesian framework, as this paradigm allows for probability statements [31], such as “There is X% probability that treatment A is the most efficacious out of all treatments”.

For treatment retention and opioid use, the treatment effect measure was the risk ratio (RR). To estimate the posterior distribution of the treatment effects, we conducted a Markov Chain Monte Carlo (MCMC) simulation with 100,000 iterations total and 5,000 burn-in iterations. To derive the posterior distribution, we ran both random-effects and fixed-effects models with an uninformative prior distribution of treatment effects. Next, we determined model fit using the deviance information criterion (DIC), where smaller DIC values correspond to better fit [32]. We then assessed the convergence of the MCMC simulations using the Gelman-Rubin-Brooks plot and the potential scale reduction factor (PSRF) [33, 34], where we determined that convergence was reached if the PSRF < 1.05.

We analyzed the outcomes by constructing the model for binary endpoints for network meta-analysis, and we calculated the RR with a 95% credible interval (CrI). To rank the preference of treatment options in the study, we plotted a rankogram and calculate the Surface Under the Cumulative Ranking (SUCRA) score [35]. The SUCRA score ranges from 0 to 1, where values closer to 1 indicate the more preferred treatments. Finally, we used the node split method to evaluate consistency of our network model, where consistency refers to the concordance of results between direct and indirect estimates within the network meta-analytic model. All analyses were conducted using R Studio version 4.0.5.

### Sensitivity analysis

To assess whether study characteristics are associated with effect size differences, we conducted univariate network meta-regression analyses by the overall risk of bias (Low Risk vs. Some Concerns or High Risk from RoB 2) and by the year of study publication (Before 2010 vs. On or After 2010). We conducted an MCMC simulation with the same conditions as above and compared model fit with the main analysis model using the DIC. We also examined the credibility of the network meta-analysis results using the Confidence in Network Meta-Analysis (CINeMA) tool to improve transparency and limit subjectivity in the evidence synthesis process [36]. For each pairwise comparison, both direct and indirect, we specifically assessed six domains: (1) within-study bias, (2) reporting bias, (3) indirectness, (4) imprecision, (5) heterogeneity, and (6) incoherence. We evaluated each domain and rated it as ‘No concerns’, ‘Some concerns’, or ‘Major concerns’ based on a set of criteria described below. For all possible pairwise comparisons across the six domains, we took the average of the level of concerns to assign a rating. For additional details, please refer to **S1 Text**.

## Results

### Search results

**Fig 1:**
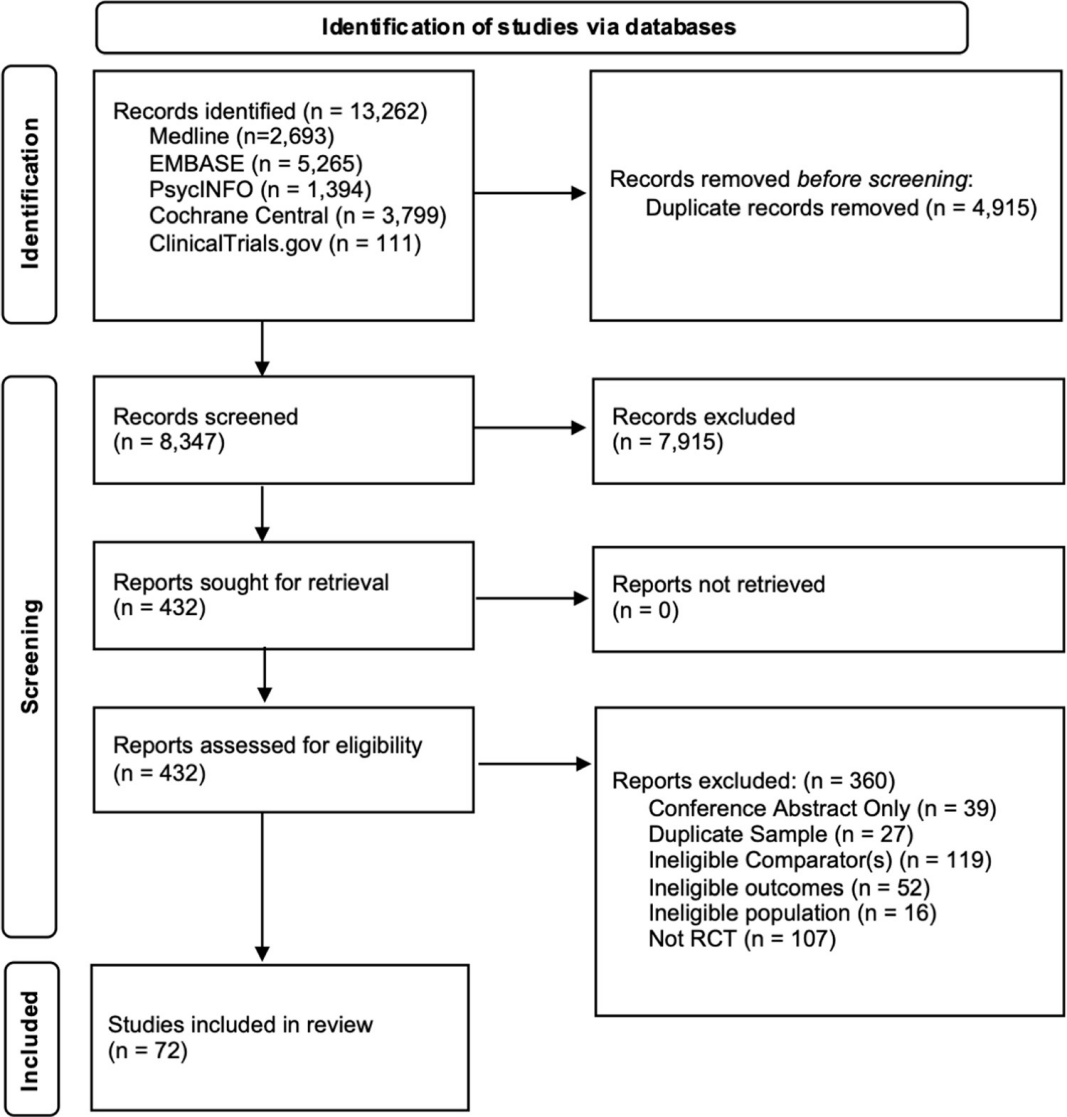
PRISMA flowchart of study selection process.

The database searches identified 13,262 publications (**Fig 1**). We removed 4,915 duplicates and an additional 7,915 studies upon review of the title and abstract. These studies were excluded because they were irrelevant to the research question or had observational study designs. Two reviewers independently examined the remaining 432 articles and excluded 360 of them (see **S4 Table** for the list of articles for full-text review and reasons for exclusion). In addition to the 72 RCTs that met our inclusion criteria, we identified 7 additional studies through grey literature search and review of earlier meta-analyses [37–43]. In total, 79 studies were included for the qualitative and quantitative evidence syntheses [37–115].

### Study characteristics

**Table 1** summarizes the key demographic and study characteristics of the RCTs included whose duration ranged from 2 weeks to 1 year. Additional details of each study can be found in the **S5 Table**. The included studies were published between 1976 and 2021, and the size of the study ranged from 19 to 1,269 participants. These trials were conducted in the United States (N = 37) [38, 39, 42, 48, 50–54, 58, 60–65, 68, 69, 71, 78–80, 82, 83, 85, 89, 90, 92, 97, 99, 101–103, 107–109, 114], Europe including Russia (N = 24) [40, 43, 47, 49, 56, 57, 66, 67, 72–77, 84, 88, 95, 96, 98, 104, 106, 110, 111, 113], Asia including the Middle East and the Caucasus (N = 14) [44–46, 59, 70, 81, 87, 91, 93, 94, 100, 105, 112, 115], and Australia (N = 4) [37, 41, 55, 86]. Most studies were financially supported by the government or non-profit organization grants. However, 10 studies did not mention their funding source [37, 41, 43, 45, 46, 48, 59, 81, 88, 104], and seven were industry-sponsored trials [47, 56, 60, 74, 82, 84, 112]. The analysis included 70 trials with a parallel design [38–46, 48–55, 57–61, 63–88, 91–99, 101–108, 110–114], three with a cross-over design [37, 47, 56], and six with a factorial design [62, 89, 90, 100, 109, 115]. There were 46 double- or triple-blind trials [38–40, 44, 48, 50, 53, 54, 56, 59–61, 63–65, 67, 68, 71, 73–77, 81–84, 86–88, 91, 92, 95–98, 100–102, 104, 105, 107–109, 111, 112], 22 open-label trials [37, 41, 43, 47, 49, 51, 52, 55, 57, 66, 69, 70, 78–80, 89, 90, 99, 103, 115], and 11 trials that did not specify blinding mechanism used [42, 45, 46, 58, 62, 72, 85, 93, 94, 106, 114].

**Table 1:** Characteristics of included studies.

First Author	Year	Journal	Country	Study Design	Blinding	Duration	Disease Condition	Interventions	Mean Age	Male %
Ahmadi	2003	Journal of Substance Abuse Treatment	Iran	Parallel	DB	18 w	Heroin dependence	BUP	BUP: 31.43	100%
								MET	MET: 33.7	
Ahmadi	2003	European Journal of Clinical Investigation	Iran	Parallel	NI	24 w	Opioid dependence	BUP	31.2	100%
								MET		
								NTX		
Ahmadi	2003	German Journal of Psychiatry	Iran	Parallel	NI	12 w	Opioid dependence	BUP	29.43	100%
								MET		
Beck	2014	Addiction	Switzerland Germany	Crossover	OL	47 w	Opioid dependence	SROM	38.1	81.5%
								MET		
Bickel	1988	Clinical Pharmacology & Therapeutics	USA	Parallel	DB	13 w	Opioid addiction	BUP	BUP: 30.2	100%
								MET	MET: 29.3	
Cameron	2006	International Journal of Pharmacy Practice	UK	Parallel	OL	12 w	Opioid dependence	BUP	NI	NI
								MET		
Clark	2006	MD Thesis Submitted to the University of Melbourne	Australia	Crossover	OL	12 w	Heroin dependence	SROM	36.5	NI
								MET		
Comer	2006	Archives of General Psychiatry	USA	Parallel	DB	8 w	Heroin dependence	NTX	41	76.70%
								CTR		
Cornish	1997	Journal of Substance Abuse Treatment	USA	Parallel	OL	6 m	Opioid addiction	NTX	39	90%
								CTR		
Coviello	2010	American Journal on Addictions	USA	Parallel	OL	6 m	Opioid dependence	NTX	33.5	82%
								CTR		
Cropsey	2011	Drug and Alcohol Dependence	USA	Parallel	DB	12 w	Opioid dependence	BUP	31.8	0%
								CTR		
Curran	1976	NIDA Research Monograph	USA	Parallel	DB	9 m	Opioid dependence	NTX	26	NI
								CTR		
D’Onofrio	2015	Journal of American Medical Association	USA	Parallel	TB	30 d	Opioid dependence	BUP	31.4	76.30%
								CTR		
Dunlop	2017	Drug and Alcohol Dependence	Australia	Parallel	OL	12 w	Heroin dependence	BNX	36.9	56%
								CTR		
Eder	2005	Addiction	Austria	Crossover	DB	14 w	Opioid dependence	SROM	SROM/MET: 29.5	87.50%
								MET	MET/SROM: 27.9	
Fischer	1999	Addiction	Austria	Parallel	OL	24 w	Opioid dependence	BUP	BUP: 25.9	68.33%
								MET	MET: 24.97	
Fudala	2003	New England Journal of Medicine	USA	Parallel	DB	4 w	Opioid dependence	BUP	BNX: 38.1	64.71%
									BUP: 36.6	
								CTR		
									CTR: 38.0	
Gruber	2008	Drug and Alcohol Dependence	USA	Parallel	NI	8.5 m	Opioid dependence	MET	MET standard: 40.2	61.26%
								CTR	MET minimal: 42.6	
									CTR: 43	
Guo	2001	Hong Kong Journal of Psychiatry	China	Parallel	DB	6 m	Opioid dependence	NTX	NTX: 24.96	NTX: 88.57%
								CTR	CTR: 26.76	Placebo: 92.86%
Haight	2019	Lancet	USA	Parallel	DB	6 m	Opioid use disorder (moderate or severe)	BUP	BUP: 39.3	BUP: 67%
										Placebo: 65%
								CTR	CTR: 39.2	
Hollister	1978	Archives of General Psychiatry	USA	Parallel	DB	9 m	Opioid dependence	NTX	NI	100%
								CTR		
Jarvis	2019	Drug and Alcohol Dependence	USA	Factorial	NI	24 w	Opioid dependence	NTX	NTX: 43.9	NTX: 60%
										UC: 83%
								CTR	UC: 42.2	
Johnson	1992	Journal of American Medical Association	USA	Parallel	DB	17 w	Heroin addiction	BUP	BUP: 33.4	BUP: 71.7%
									MET 20 mg: 32.7	MET 20 mg: 69.1%
								MET		
									MET 60 mg: 33.1	MET 60 mg: 68.5%
Johnson	1995	Drug and Alcohol Dependence	USA	Parallel	DB	2 w	Opioid dependence	BUP	BUP 8 mg: 31.65	BUP 8 mg: 50%
									BUP 2 mg: 33.70	BUP 2 mg: 75%
								CTR		
									CTR: 34.99	Placebo: 72%
Johnson	2000	New England Journal of Medicine	USA	Parallel	DB	17 w	Opioid dependence	BUP	BUP: 36	BUP: 66%
										MET: 64%
								MET	MET: 36	
Kakko	2003	Lancet	Sweden	Parallel	OL	1 y	Heroin dependence	BUP	BUP: 29.2	72.50%
								CTR	CTR:31.5	
Kakko	2007	American Journal of Psychiatry	Sweden	Parallel	DB	6 m	Heroin dependence	BUP	BUP: 34.8	79.20%
								MET	MET: 36.5	
Kamien	2008	Heroin Addiction & Related Health Problems	USA	Parallel	DB	17 w	Opioid dependence	BNX	BNX 8/2: 37.2	71.27%
									BNX 16/4: 38.9	
								MET		
									MET 45: 40.3	
									MET 90: 38.1	
Kinlock	2009	Journal of Substance Abuse Treatment	USA	Parallel	OL	12 m	Heroin dependence	MET	40.3	100%
								CTR		
Korthuis	2021	Lancet HIV	Vietnam	Parallel	OL	12 m	Opioid use disorder (moderate or severe)	BNX	38.3	97%
								MET		
Kosten	1993	Journal of Nervous and Mental Disease	USA	Parallel	DB	6 m	Opioid dependence	BUP	32	73%
								MET		
Kristensen	2005	Tidsskrift for Den norske legeforening	Norway	Parallel	NI	26 w	Opioid addiction	BUP	BUP: 36	76%
								MET	MET: 36	
Krook	2002	Addiction	Norway	Parallel	DB	12 w	Opioid dependence	BUP	38	66.04%
								CTR		
Krupitsky	2004	Journal of Substance Abuse Treatment	Russia	Parallel	DB	6 m	Heroin dependence	NTX	NTX: 22.8	80.80%
								CTR	CTR: 20.7	
Krupitsky	2006	Journal of Substance Abuse Treatment	Russia	Parallel	DB	6 m	Opioid dependence	NTX	NTX: 23.9	NTX: 73%
										CTR: 64%
								CTR	CTR: 23.6	
Krupitsky	2011	Lancet	Russia	Parallel	DB	24 w	Opioid dependence	NTX	NTX: 29.4	88%
								CTR	CTR: 29.7	
Krupitsky	2012	Archives of General Psychiatry	Russia	Parallel	DB	24 w	Opioid dependence	NTX	28.2	72.50%
								CTR		
Krupitsky	2013	Drug and Alcohol Dependence	Russia	Parallel	DB	6 m	Opioid dependence	NTX	28.3	82.40%
								CTR		
Lee	2015	Addiction	USA	Parallel	OL	8 w	Opioid dependence	NTX	NTX: 40	100%
								CTR	CTR: 47	
Lee	2016	New England Journal of Medicine	USA	Parallel	OL	24 w	Opioid dependence	NTX	44	85%
								CTR		
Lee	2018	Lancet	USA	Parallel	OL	24 w	Opioid use disorder	NTX	NTX: 34.0	70.35%
								BNX	BNX: 33.7	
Lerner	1992	Israel Journal of Psychiatry and Related Sciences	Israel	Parallel	DB	2 m	Opioid addiction	NTX	26.6	NI
								CTR		
Ling	1996	Archives of General Psychiatry	USA	Parallel	DB	52 w	Opioid dependence	BUP	40.8	80%
								MET		
Ling	2010	Journal of American Medical Association	USA	Parallel	DB	6 m	Opioid dependence	BUP	BUP: 35.8	68.71%
								CTR	CTR: 39.3	
Lintzeris	2004	American Journal on Addictions	Australia	Parallel	OL	12 m	Heroin dependence	BUP	NI	NI
								MET		
Madlung-Kratzer	2009	Addiction	Austria	Parallel	DB	22 d	Opioid addiction	SROM	SROM: 27.4	75.25%
								MET	MET: 28.2	
Magura	2009	Drug and Alcohol Dependence	USA	Parallel	NI	3 m	Opioid dependence	BUP	BUP: 38.4	100%
								MET	MET: 40.7	
Mattick	2003	Addiction	Australia	Parallel	DB	13 w	Opioid dependence	BUP	30	69.38%
								MET		
Mokri	2016	Addiction	Iran	Parallel	DB	12 w	Opioid dependence	NTX	28.6	NI
								BNX		
Neri	2005	Psychopharmacology	Italy	Parallel	DB	12 m	Opioid addiction	BUP	25	88.71%
								MET		
Neumann	2013	Journal of Addictive Diseases	USA	Factorial	OL	6 m	Opioid dependence	BNX	38.3	53.70%
								MET		
Neumann	2020	Journal of Addictive Diseases	USA	Factorial	OL	6 m	Opioid dependence	BNX	NI	31.58%
								MET		
Newman	1979	Lancet	Hong Kong	Parallel	DB	3 y	Heroin addiction	MET	MET: 38	100%
								CTR	CTR: 38	
Oliveto	1999	Archives of General Psychiatry	USA	Parallel	DB	13 w	Opioid dependence	BUP	BUP: 34.3	68.89%
								MET	MET: 33.9	
Otiashvili	2012	Drug and Alcohol Dependence	Republic of Georgia	Parallel	NI	22 w	Opioid dependence	NTX	35.6	100%
								CTR		
Otiashvili	2013	Drug and Alcohol Dependence	Republic of Georgia	Parallel	NI	12 w	Opioid dependence	BNX	33.7	95%
								MET		
Pani	2000	Drug and Alcohol Dependence	Italy	Parallel	DB	6 m	Opioid dependence	BUP	BUP: 28	86.11%
								MET	MET: 28	
Petitjean	2001	Drug and Alcohol Dependence	Switzerland	Parallel	DB	6 w	Opioid dependence	BUP	27.3	82.76%
								MET		
Rosenthal	2013	Addiction	USA	Parallel	DB	24 w	Opioid dependence	BUP	BI: 36.4	BI: 63.2%
									SB: 35.3	SB: 57.4%
								CTR	CTR: 35.2	CTR: 60.5%
San	1991	British Journal of Addiction	Spain	Parallel	DB	6 m	Opioid (heroin) dependence	NTX	NTX: 26.1	76%
								CTR	CTR: 27.3	
Saxon	2013	Drug and Alcohol Dependence	USA	Parallel	OL	32 w	Opioid dependence	BUP	BUP: 39.3	BUP: 71.2%
								MET	MET: 38.4	MET: 64.7%
Schottenfeld	1997	Archives of General Psychiatry	USA	Parallel	DB	24 w	Opioid dependence Cocaine dependence	BUP	BUP 4 mg: 33.7	68.97%
									BUP 12 mg: 32.6	
								MET		
									MET 20 mg: 31.6	
									MET 65 mg: 32.6	
Schottenfeld	2005	American Journal of Psychiatry	USA	Parallel	DB	24 w	Opioid dependence	BUP	36.2	66%
							Cocaine dependence	MET		
							Cocaine abuse			
Schottenfeld	2008	Lancet	Malaysia	Factorial	DB	24 w	Opioid dependence	NTX	NTX: 38.2	NI
								BUP	BUP: 36.3	
								CTR	CTR: 37.6	
Schottenfeld	2021	Addiction	Malaysia	Factorial	OL	24 w	Opioid dependence	BUP	38.7	100%
								CTR		
Schwartz	2006	Archives of General Psychiatry	USA	Parallel	OL	4 m	Heroin dependence	MET	41.4	59.20%
								CTR		
Schwartz	2020	Drug and Alcohol Dependence	USA	Parallel	NI	12 m	Opioid use disorder	MET	38.3	80.40%
								CTR		
Seifert	2002	Pharmacopsychiatry	Germany	Parallel	DB	14 d	Opioid dependence	BUP	BUP: 31.1	84.62%
								MET	MET: 31.8	
Shufman	1994	Biological Psychiatry	Israel	Parallel	DB	12 w	Opioid dependence	NTX	NTX: 33.6	100%
								CTR	CTR: 32	
Soyka	2008	International Journal of Neuropsychopharmacology	Germany	Parallel	NI	6 m	Opioid dependence	BUP	BUP: 31.2	65.71%
								MET	MET: 27.9	
Stella	2005	Life Sciences	Italy	Parallel	OL	6 m	Opioid dependence	NTX	NI	91.07%
								CTR		
Strain	1994	Psychopharmacology	USA	Parallel	DB	26 w	Opioid dependence	BUP	32	71%
								MET		
Strain	1994	American Journal of Psychiatry	USA	Parallel	DB	26 w	Opioid dependence	BUP	BUP: 32.2	70.73%
								MET	MET: 32.8	
Sullivan	2015	Drug and Alcohol Dependence	USA	Factorial	DB	24 w	Opioid dependence	NTX	BNT+XR-NTX: 38.1	78.65%
									BNT+CTR: 40.1	
								CTR		
									CE+XR-NTX: 37.3	
									CE+CTR: 36.7	
Tanum	2017	JAMA Psychiatry	Norway	Parallel	OL	12 w	Opioid dependence	NTX	NTX: 36.4	72.33%
								BNX	BNX: 35.7	
Tiihonen	2012	American Journal of Psychiatry	Russia	Parallel	DB	10 w	Opioid dependence	NTX	NTX: 28.0	89%
								CTR	CTR: 29.3	
Wang	2018	Asia-Pacific Psychiatry	China	Parallel	DB	10 w	Opioid dependence	BNX	BNX: 43.92	81.92%
								CTR	CTR: 44.40	
Wright	2011	British Journal of General Practice	UK	Parallel	OL	6 m	Opioid dependence	BUP	BUP: 31.0	NI
								MET	MET: 30.7	
Yancovitz	1991	American Journal of Public Health	USA	Parallel	NI	1 m	Heroin addiction	MET	MET: 34.8	79.40%
								CTR	CTR: 34.4	

Abbreviations: BI: buprenorphine implants, BUP: buprenorphine, BNX: buprenorphine-naloxone, CE: compliance enhancement, CTR: control (includes standard of care, usual care, treatment as usual, behavioural counselling, and placebo), MET: methadone, NTX: naltrexone, XR-NTX: extended-release naltrexone, SB: sublingual buprenorphine, SROM: slow-release oral morphine, NI: no information; DB: double-blind, TB: triple-blind, OL: open label, d: days, w: weeks, m: months, y: years.

Most of the patients in the included studies were male (78% among those that reported it for the entire study population before randomization), with 12 trials including only male participants [44–46, 48, 61, 69, 79, 85, 91, 93, 105, 115]. The mean age of the participants in the study was 34 among those that reported it for the entire study population before randomization (range of mean age: 20 to 47). All of the included studies focused on patients with OUD, with 13 studies focusing specifically on patients with heroin-related disorders [37, 41, 44, 50, 55, 65–67, 69, 77, 91, 103, 114]. Two studies recruited patients with co-occurring opioid use disorder and chronic non-cancer pain [89, 90], while 61 studies excluded patients with psychiatric disorders or concurrent dependence on alcohol, sedative, or hypnotic medications [37, 39, 40, 42, 43, 48–50, 52, 54–57, 59, 60, 62–68, 70, 74–80, 82–88, 90–93, 95–113, 115].

### Risk of bias of included RCTs

Overall, the risk of bias was judged ‘Low’ for 38 studies [39, 40, 50, 53–57, 60, 63–67, 70, 73–80, 82, 83, 85–87, 89, 90, 97, 100–102, 109–112], ‘Some Concerns’ for 37 studies [37, 38, 41–43, 45–49, 51, 52, 58, 59, 62, 68, 71, 72, 81, 84, 88, 91–96, 98, 99, 104–108, 113, 115], and ‘High’ for 4 studies [44, 69, 103, 114] (**Table 2**; see **S6 Table** for a more detailed assessment of risk of bias). For randomization and allocation concealment, 49 trials were judged ‘Low Risk’ [39–41, 48, 50, 53–57, 59–61, 63–68, 70, 71, 73–80, 82, 83, 85–87, 89, 90, 92, 96–98, 100–102, 104, 109–113], and 30 trials were judged ‘Some Concerns’ [37, 38, 42–47, 49, 51, 52, 58, 62, 69, 72, 81, 84, 88, 91, 93–95, 99, 103, 105–108, 114, 115]. The trials in the latter group were rated as such due to unclear reporting of whether the allocation sequence was concealed until participants were enrolled and assigned to interventions. That is, these studies provided no information on whether treatment allocation was remotely or centrally administered. For the three cross-over studies [37, 47, 56], we also examined the risk of bias associated with period or carryover effects. Two studies [47, 56] were judged to have ‘Low Risk’ of carryover effects, but one study [37] was judged to have ‘Some Concerns’ because it had provided no information on whether there was sufficient time between the two study periods for the carryover effects to have disappeared. For most studies, the risk of bias due to deviations from the intended interventions and the risk of bias due to missing outcome data was ‘Low’. However, two trials were judged to have higher risk of bias due to deviations from the intended interventions because a large number of patients failed to remain and adhere to the originally assigned treatment shortly after randomization [44, 114]. Relatedly, four studies that examined opioid use as their main endpoint were at higher risk of bias due to missing outcome data because they employed per-protocol analyses that excluded participants who were lost to follow-up before the end of the study period [43, 69, 103, 114]. Since both treatment retention and opioid use were measured objectively, the risk of bias in the measurement of outcome was judged ‘Low Risk’ for all studies, and the risk of bias from selective reporting remains low. However, due to the lack of available protocol in the grant (e.g., NIH REPORTER) or trial registry (e.g., ClinicalTrials.gov), the risk of reporting bias was judged ‘Some Concerns’ for 31 studies [37, 38, 41, 43–46, 48, 49, 52, 58, 59, 61, 68, 71, 72, 81, 84, 88, 91, 92, 95, 96, 98, 99, 103–105, 107, 108, 113].

**Table 2:** Risk of bias assessment.

Study	Randomization	Effect of Assignment to Intervention	Effect of Adherence to Intervention	Missing Outcome Data	Measurement of Outcome	Selection of Reported Result	Period or Carryover Effects	Overall Risk of Bias
Ahmadi 2003a	Some Concerns	Low	High	Low	Low	Some Concerns		High
Ahmadi 2003b	Some Concerns	Low	Low	Low	Low	Some Concerns		Some Concerns
Ahmadi 2003c	Some Concerns	Low	Low	Low	Low	Some Concerns		Some Concerns
Beck 2014	Some Concerns	Low	Low	Low	Low	Low	Low	Some Concerns
Bickel 1988	Low	Low	Low	Low	Low	Some Concerns		Some Concerns
Cameron 2006	Some Concerns	Low	Low	Low	Low	Some Concerns		Some Concerns
Clark 2006	Some Concerns	Low	Low	Low	Low	Some Concerns	Some Concerns	Some Concerns
Comer 2006	Low	Low	Low	Low	Low	Low		Low
Cornish 1997	Some Concerns	Low	Low	Low	Low	Low		Some Concerns
Coviello 2010	Some Concerns	Low	Low	Low	Low	Some Concerns		Some Concerns
Cropsey 2011	Low	Low	Low	Low	Low	Low		Low
Curran 1976	Some Concerns	Low	Low	Low	Low	Some Concerns		Some Concerns
D’Onofrio 2015	Low	Low	Low	Low	Low	Low		Low
Dunlop 2017	Low	Low	Low	Low	Low	Low		Low
Eder 2005	Low	Low	Low	Low	Low	Low	Low	Low
Fischer 1999	Low	Low	Low	Low	Low	Low		Low
Fudala 2003	Low	Low	Low	Low	Low	Low		Low
Gruber 2008	Some Concerns	Low	Low	Low	Low	Some Concerns		Some Concerns
Guo 2001	Low	Low	Low	Low	Low	Some Concerns		Some Concerns
Haight 2019	Low	Low	Low	Low	Low	Low		Low
Hollister 1978	Low	Low	Low	Low	Low	Some Concerns		Some Concerns
Jarvis 2019	Some Concerns	Low	Low	Low	Low	Low		Some Concerns
Johnson 1992	Low	Low	Low	Low	Low	Low		Low
Johnson 1995	Low	Low	Low	Low	Low	Low		Low
Johnson 2000	Low	Low	Low	Low	Low	Low		Low
Kakko 2003	Low	Low	Low	Low	Low	Low		Low
Kakko 2007	Low	Low	Low	Low	Low	Low		Low
Kamien 2008	Low	Low	Low	Low	Low	Some Concerns		Some Concerns
Kinlock 2009	Some Concerns	Some Concerns	Low	High	Low	Low		High
Korthuis 2021	Low	Low	Low	Low	Low	Low		Low
Kosten 1993	Low	Low	Low	Low	Low	Some Concerns		Some Concerns
Kristensen 2005	Some Concerns	Low	Low	Low	Low	Some Concerns		Some Concerns
Krook 2002	Low	Low	Low	Low	Low	Low		Low
Krupitsky 2004	Low	Low	Low	Low	Low	Low		Low
Krupitsky 2006	Low	Low	Low	Low	Low	Low		Low
Krupitsky 2011	Low	Low	Low	Low	Low	Low		Low
Krupitsky 2012	Low	Low	Low	Low	Low	Low		Low
Krupitsky 2013	Low	Low	Low	Low	Low	Low		Low
Lee 2015	Low	Low	Low	Low	Low	Low		Low
Lee 2016	Low	Low	Low	Low	Low	Low		Low
Lee 2018	Low	Low	Low	Low	Low	Low		Low
Lerner 1992	Some Concerns	Low	Low	Low	Low	Some Concerns		Some Concerns
Ling 1996	Low	Low	Low	Low	Low	Low		Low
Ling 2010	Low	Low	Low	Low	Low	Low		Low
Lintzeris 2004	Low	Low	Low	Low	Low	Some Concerns		Some Concerns
Madlung-Kratzer 2009	Some Concerns	Low	Low	Low	Low	Some Concerns		Some Concerns
Magura 2009	Low	Low	Low	Low	Low	Low		Low
Mattick 2003	Low	Low	Low	Low	Low	Low		Low
Mokri 2016	Low	Low	Low	Low	Low	Low		Low
Neri 2005	Some Concerns	Low	Low	Low	Low	Some Concerns		Some Concerns
Neumann 2013	Low	Low	Low	Low	Low	Low		Low
Neumann 2020	Low	Low	Low	Low	Low	Low		Low
Newman 1979	Some Concerns	Low	Low	Low	Low	Some Concerns		Some Concerns
Oliveto 1999	Low	Low	Low	Low	Low	Some Concerns		Some Concerns
Otiashvili 2012	Some Concerns	Low	Low	Low	Low	Low		Some Concerns
Otiashvili 2013	Some Concerns	Low	Low	Low	Low	Low		Some Concerns
Pani 2000	Some Concerns	Low	Low	Low	Low	Some Concerns		Some Concerns
Petitjean 2001	Low	Low	Low	Low	Low	Some Concerns		Some Concerns
Rosenthal 2013	Low	Low	Low	Low	Low	Low		Low
San 1991	Low	Low	Low	Low	Low	Some Concerns		Some Concerns
Saxon 2013	Some Concerns	Low	Low	Low	Low	Some Concerns		Some Concerns
Schottenfeld 1997	Low	Low	Low	Low	Low	Low		Low
Schottenfeld 2005	Low	Low	Low	Low	Low	Low		Low
Schottenfeld 2008	Low	Low	Low	Low	Low	Low		Low
Schottenfeld 2021	Some Concerns	Some Concerns	Low	Low	Low	Low		Some Concerns
Schwartz 2006	Some Concerns	Some Concerns	Low	High	Low	Some Concerns		High
Schwartz 2020	Some Concerns	Low	Low	Low	Low	Low		Some Concerns
Seifert 2002	Low	Low	Low	Low	Low	Some Concerns		Some Concerns
Shufman 1994	Some Concerns	Low	Low	Low	Low	Some Concerns		Some Concerns
Soyka 2008	Some Concerns	Low	Low	Low	Low	Low		Some Concerns
Stella 2005	Some Concerns	Some Concerns	Low	Some Concerns	Low	Some Concerns		Some Concerns
Strain 1994a	Some Concerns	Low	Low	Low	Low	Some Concerns		Some Concerns
Strain 1994b	Some Concerns	Low	Low	Low	Low	Some Concerns		Some Concerns
Sullivan 2015	Low	Low	Low	Low	Low	Low		Low
Tanum 2017	Low	Low	Low	Low	Low	Low		Low
Tiihonen 2012	Low	Low	Low	Low	Low	Low		Low
Wang 2018	Low	Low	Low	Low	Low	Low		Low
Wright 2011	Low	Low	Low	Low	Low	Some Concerns		Some Concerns
Yancovitz 1991	Some Concerns	High	High	High	Low	Low		High

### Evidence synthesis

Due to differences in approach to measurement of opioid use (e.g., differences in defining abstinence) even between studies using urinalysis to ascertain it, we judged that the network meta-analysis (NMA) would not produce reliable estimates for the secondary outcome. Therefore, we only conducted NMA for treatment retention (primary outcome).

#### Synthesis of primary outcome

**Fig 2:**
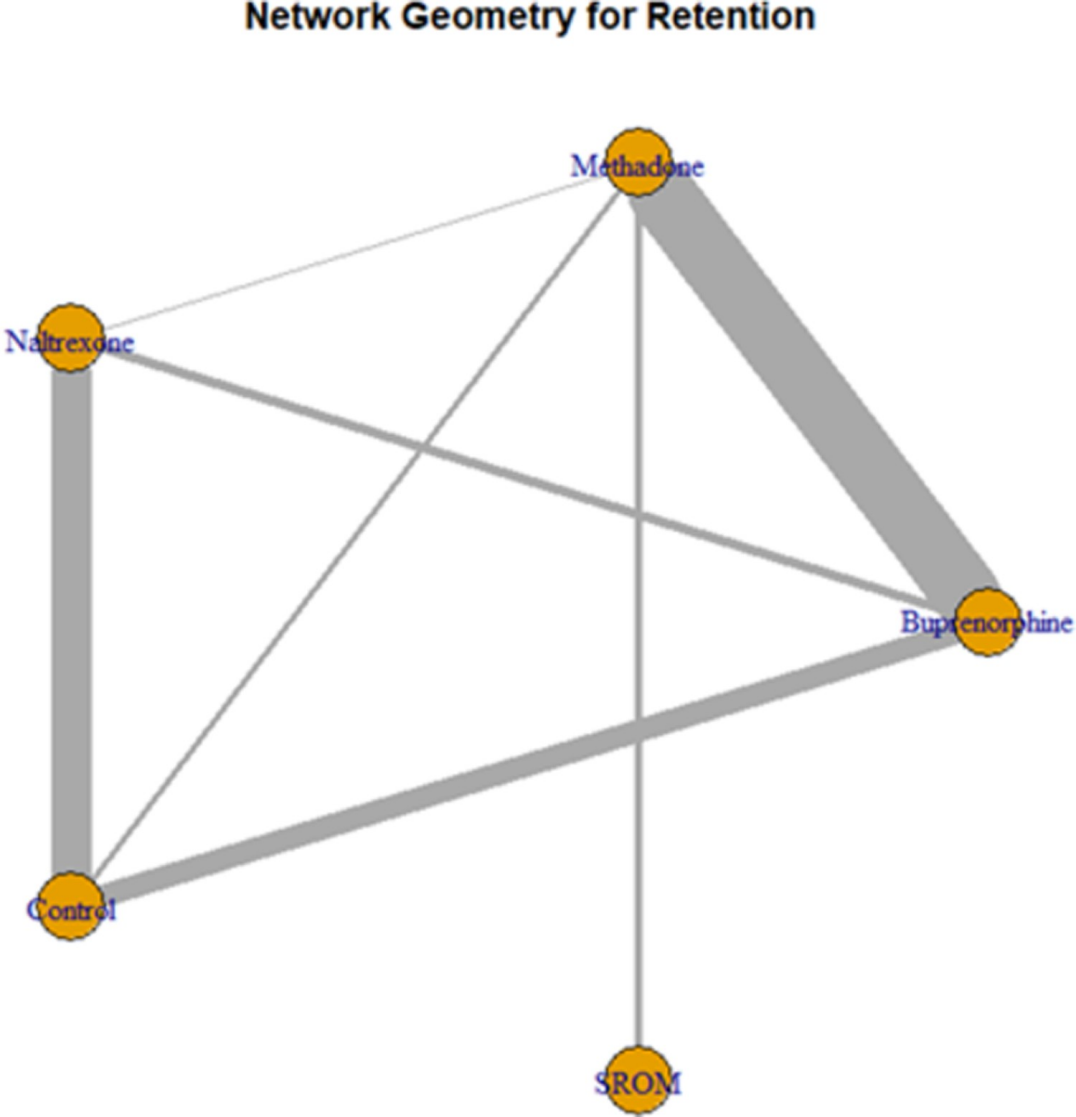
Network geometry for the treatment retention outcome for each medication or control group.

In our network meta-analysis, 73 studies, representing 5 interventions, presented data on treatment retention [37–42, 44–61, 63–68, 70–102, 104–113], measured as the number of patients who remained in treatment at the end of the study. **Fig 2** illustrates a network diagram that summarizes the overall structure of the competing treatments in our network of interventions. The most frequently analyzed pairwise comparison has been the buprenorphine-methadone comparison (N = 35 from 32 studies) [41, 44–46, 48, 49, 57, 63, 65, 67, 68, 70–72, 83, 85, 86, 88–90, 92, 94–96, 99, 101, 102, 104, 106–108, 113], followed by the naltrexone-control (N = 21) [38, 40, 50–52, 59, 61, 62, 74–79, 81, 93, 98, 100, 105, 109, 111] and buprenorphine-control (N = 13) [39, 53–55, 60, 64, 66, 73, 82, 97, 100, 112, 115] comparisons. While treatment retention for methadone has been compared with that for all other treatments and controls in our network, the efficacy of SROM has only been compared with that of methadone [37, 47, 56, 84].

For the primary analysis, we initially ran both fixed effects (FE) and random effects (RE) model. Upon comparison of the DIC values, both models demonstrated similar model fit (FE: 294.28 vs. RE: 294.58), but we chose to conduct all subsequent analyses with the random effects model (see **S7 Table** for the explanation and model fit comparison). The MCMC simulation with the random effects model resulted in the PSRF of 1.0037, suggesting convergence of the MCMC algorithm (i.e., the simulation resulted in an accurate estimate of our parameters; see **S1** and **S2 Figs** for detailed results of the simulation).

**Fig 3:**
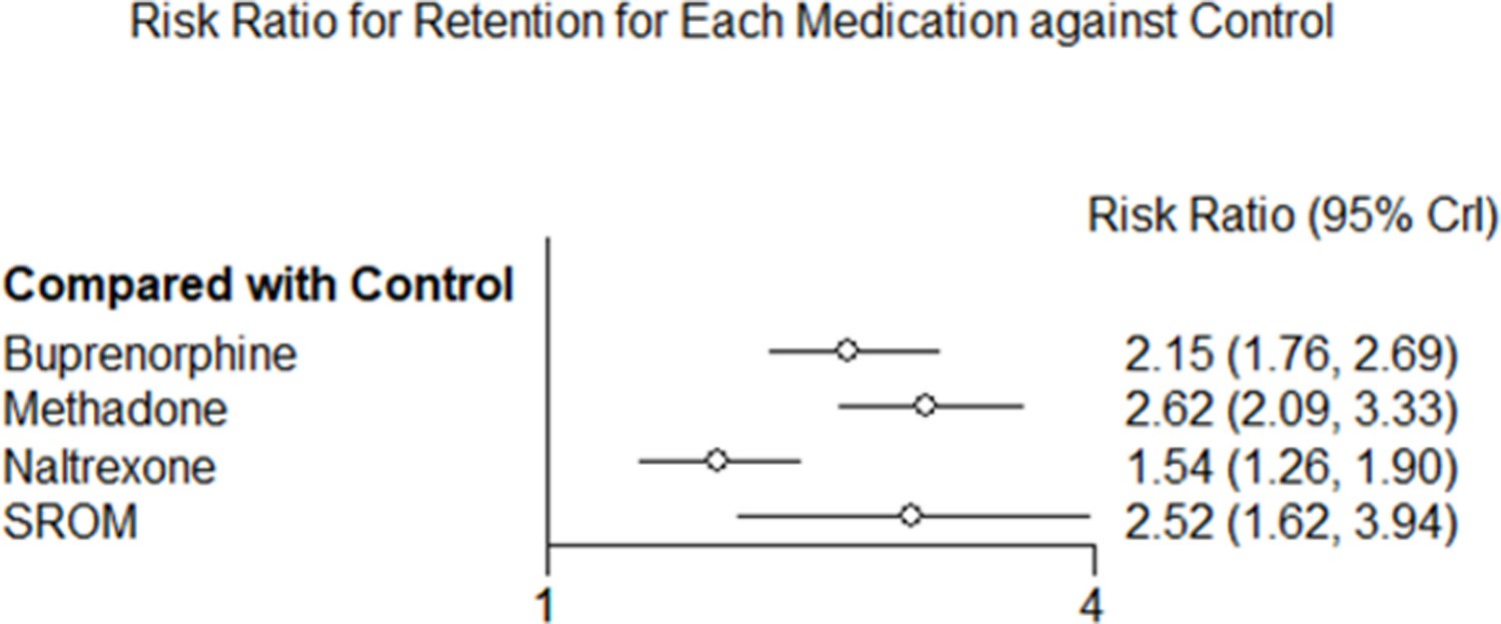
Forest plot of the risk ratio for treatment retention for each pharmacotherapy compared to the control group.

All pharmacotherapy options were more efficacious with respect to treatment retention than the control group. Compared to the control group, the likelihood of retention was 2.62 (95% CrI = 2.09–3.33), 2.52 (95% CrI = 1.62–3.94), 2.15 (95% CrI = 1.76–2.69), and 1.54 (95% CrI = 1.26–1.90) times higher for methadone, SROM, buprenorphine, and naltrexone, respectively (**Fig 3**). Further, the average percentage of treatment retention across all studies was 77.6% for SROM, 64.1% for methadone, 54.3% for buprenorphine, 41.0% for naltrexone, and 30.1% for control (see **S8 Table** and **S3 Fig**). Analysis using the SUCRA score also indicated similar findings (see **S8 Table** and **S4 Fig** for additional details). Methadone was the highest ranked treatment based on the SUCRA value of 0.901. The next highest ranked treatment was SROM (SUCRA = 0.784), followed by buprenorphine (SUCRA = 0.559), naltrexone (SUCRA = 0.257), and control (SUCRA = 0.000). While the SUCRA values demonstrated which treatment in the network may be the most efficacious, pairwise comparisons revealed the relative effectiveness of one treatment versus the other in retaining patients in the initially assigned treatment (**Table 3**). Methadone had a higher likelihood of retention than buprenorphine (RR = 1.22; 95% CrI = 1.06–1.40) and naltrexone (RR = 1.69; 95% CrI = 1.30–2.24) but remained statistically equivalent to SROM (RR = 1.04; 95% CrI = 0.71–1.52). Similarly, buprenorphine (RR = 1.39; 95% CrI = 1.10–1.80) and SROM (RR = 1.63; 95% CrI = 1.01–2.63) both had a higher likelihood of retention than naltrexone, but the two medications remained equivalent (RR = 0.86; 95% CrI = 0.57–1.28 with SROM as the reference group).

**Table 3:** Risk ratio estimates for treatment retention and 95% credible interval (CrI)^1^.

	Buprenorphine	Methadone	Naltrexone	Control	SROM
**Buprenorphine**	Buprenorphine	1.22 (1.06, 1.40)	0.72 (0.55, 0.91)	0.47 (0.37, 0.57)	1.17 (0.78, 1.74)
**Methadone**	0.82 (0.71, 0.95)	Methadone	0.59 (0.45, 0.77)	0.38 (0.30, 0.48)	0.96 (0.66, 1.4)
**Naltrexone**	1.39 (1.10, 1.80)	1.69 (1.30, 2.24)	Naltrexone	0.65 (0.53, 0.79)	1.63 (1.01, 2.63)
**Control**	2.15 (1.76, 2.69)	2.62 (2.09, 3.33)	1.54 (1.26, 1.9)	Control	2.52 (1.62, 3.94)
**SROM**	0.86 (0.57, 1.28)	1.04 (0.71, 1.52)	0.62 (0.38, 0.99)	0.4 (0.25, 0.62)	SROM

^1^ When read horizontally, the risk ratio estimates are those from comparing a treatment group with the drug name in the cell. For example, the risk ratio (RR) of 1.22 (95% CrI = 1.06–1.40) refers to the relative risk of retention associated with methadone compared to buprenorphine as the reference group. Similarly, the RR = 0.72 (95% CrI = 0.55–0.91) refers to the naltrexone versus buprenorphine comparison, where the latter was the reference group. When read vertically, the drug name in the left-most column is the reference group in a comparison with the medication listed in the top row.

**Fig 4:**
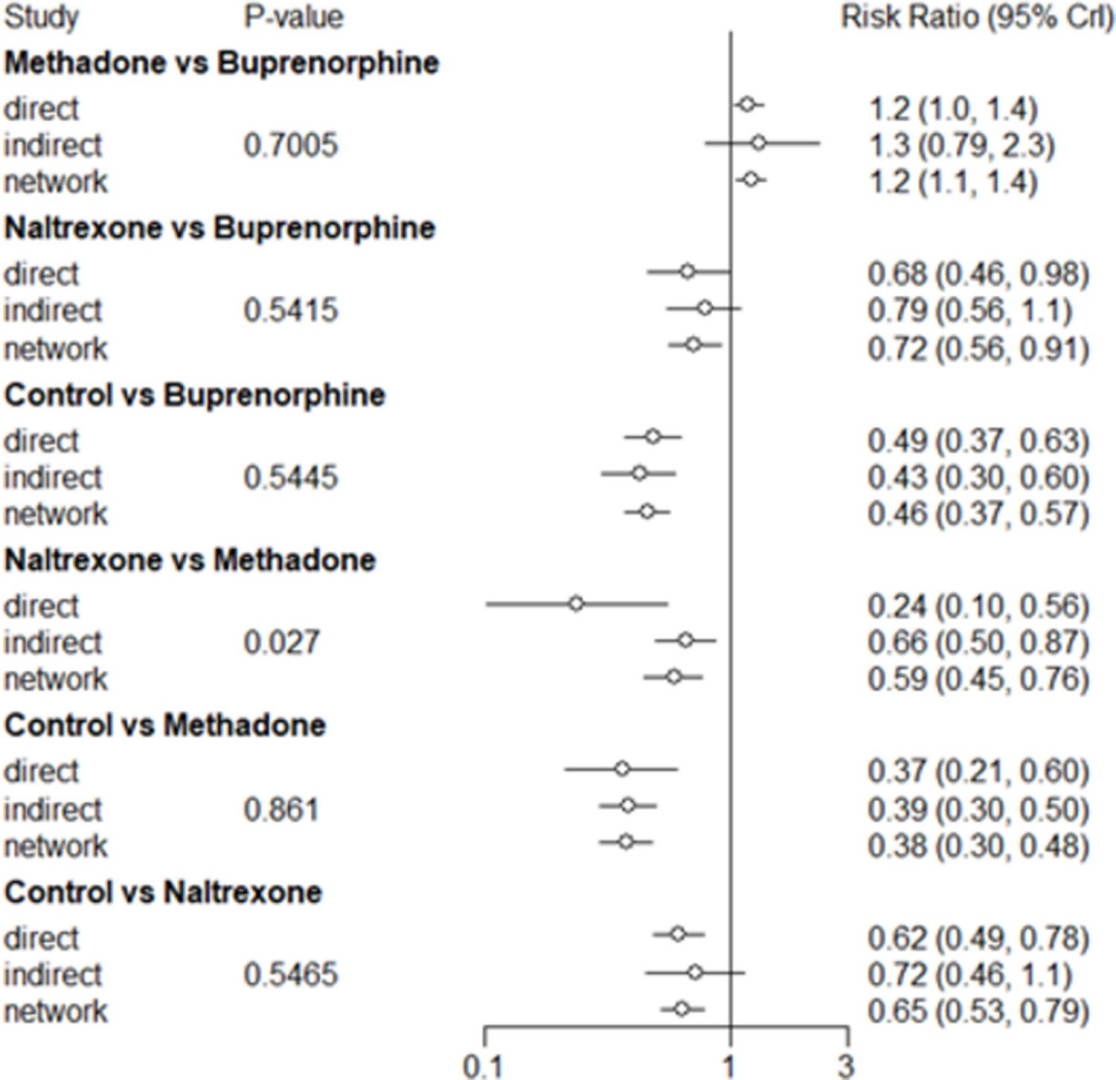
Direct and indirect estimate of the likelihood of retention between treatments using the node-splitting method to assess consistency of the network model.

**Fig 4** illustrates forest plots of direct and indirect estimates of likelihood of treatment retention from the node split method, which was used to evaluate consistency of our network model. Results from six pairwise comparisons involving buprenorphine, methadone, naltrexone, and control group were presented. Pairwise comparisons involving SROM were not included in this node split approach because methadone was its only comparator, and SROM was disconnected from all other interventions including the control group in the network graph. For all pairwise comparisons except naltrexone versus methadone, the estimates from direct and indirect comparisons were statistically equivalent (p-value > 0.05). However, for the naltrexone versus methadone pair, there was some evidence of inconsistency (p-value = 0.027), as the magnitude of the RR was larger for the direct estimate than for the indirect estimate.

As for sensitivity analysis, model fit from univariate network meta-regression analysis adjusting for risk of bias and publication year remained similar to that from the unadjusted analysis (DIC: Unadjusted = 294.58; Risk of bias = 293.46; Publication year = 294.66). Thus, it remains unlikely that controlling for these study characteristics and methodological differences between studies influenced the magnitude of the effect sizes in our network. Using the CINeMA tool (**Table 4, S5** and **S6 Figs**), we judged with ‘High’ level of confidence the estimates of the likelihood of treatment retention comparing buprenorphine vs. control and methadone vs. control. For the buprenorphine vs. methadone and buprenorphine vs. naltrexone comparisons, we assigned a ‘Moderate’ confidence rating due to the presence of heterogeneity. For the methadone vs. naltrexone comparison, we assigned a confidence rating of ‘Low’ due to having a small number of studies as well as concerns related to the ‘Heterogeneity’ and ‘Incoherence’ domains for the estimates derived from the network. Finally, all pairwise comparisons involving SROM received the confidence rating of ‘Very Low’ due to high degree of incoherence that resulted from insufficient evidence in the network.

**Table 4:** Summary of credibility of the network estimates using the Confidence in Network Meta-Analysis (CINeMA) tool.

Comparison	Number of studies	Within-study bias	Reporting bias	Indirectness	Imprecision	Heterogeneity	Incoherence	Confidence rating	Reason(s) for downgrading
Buprenorphine-Control	12	No concerns	Low risk	No concerns	No concerns	No concerns	No concerns	High	[]
Buprenorphine-Methadone	35	Some concerns	Low risk	No concerns	No concerns	Some concerns	No concerns	Moderate	["Heterogeneity"]
Buprenorphine-Naltrexone	5	No concerns	Low risk	No concerns	No concerns	Some concerns	No concerns	Moderate	["Heterogeneity"]
Methadone-Control	3	Some concerns	Low risk	No concerns	No concerns	No concerns	No concerns	High	[]
Naltrexone-Control	21	No concerns	Low risk	No concerns	No concerns	Some concerns	No concerns	Moderate	["Heterogeneity"]
Methadone-Naltrexone	1	Some concerns	Some concerns	No concerns	No concerns	Some concerns	Some concerns	Low	["Reporting bias", "Heterogeneity", "Incoherence"]
Methadone-SROM	4	Some concerns	Some concerns	No concerns	No concerns	Major concerns	Major concerns	Very low	["Reporting bias", "Heterogeneity", "Incoherence"]
Buprenorphine-SROM	0	Some concerns	Low risk	No concerns	Some concerns	Some concerns	Major concerns	Very low	["Incoherence"]
SROM-Control	0	Some concerns	Low risk	No concerns	No concerns	No concerns	Major concerns	Very low	["Incoherence"]
Naltrexone-SROM	0	Some concerns	Low risk	No concerns	No concerns	Some concerns	Major concerns	Very low	["Incoherence"]

#### Synthesis of secondary outcomes

Due to both small number of studies reporting secondary outcomes and inconsistent reporting formats, we were unable to meta-analyze studies reporting these outcomes. We present below details of the post-hoc analysis of the secondary outcomes.

Thirty-five studies [47, 50–53, 57, 59–63, 65, 71, 72, 76, 78, 79, 82, 83, 88, 93–98, 101, 102, 107, 108, 110–112, 115] assessed opioid use as the percentage of opioid positive urine samples; twelve studies [43, 52, 53, 61, 62, 69, 74, 87, 98, 103, 105, 114] assessed opioid use as the number of patients who had at least one opioid positive urine sample at the end of the study out of all those randomized to each arm in the beginning of the study; and three studies [61, 62, 98] applied both definitions. In addition, there were heterogeneities in reporting opioid use across studies. Among the studies that reported percentage of opioid positive urine samples, nine reported the total number of tests and number of positive urine samples [50, 59, 61, 62, 76, 79, 82, 88, 94], eight reported percentages with a 95% CI or standard error [47, 50, 54, 63, 76, 82, 97, 115], and six reported percentages with a standard deviation [60, 62, 83, 95, 101, 110]. However, 13 studies only reported the percentage value without other parameters to assess variability of the sample [51, 57, 65, 71, 72, 78, 96, 98, 102, 107, 108, 111, 112].

### Discussion

All pharmacotherapeutic strategies were associated with a higher likelihood of treatment retention compared to the control arm, which consisted of placebo, standard of care, or no treatment. Based on SUCRA rankings, methadone appeared to be the most effective pharmacotherapy for treatment retention, followed by SROM, buprenorphine, and naltrexone. Buprenorphine was superior to naltrexone, and naltrexone was superior to non-pharmacotherapeutic interventions in treatment retention. SROM was compared only with methadone, and all other pairwise comparisons involving SROM were based on indirect evidence. The lack of available evidence on direct comparison also raised major concerns with respect to the ‘Incoherence’ domain in the CINeMA, which in turn lowered confidence in the estimates related to SROM generated by the NMA.

To our knowledge, this is the first network meta-analysis of the medications for opioid use disorder to examine their relative efficacy against each other. The findings from this study remain consistent with those from earlier meta-analyses by Mattick et al. (2014) and Mattick et al. (2009) [24, 25], which concluded that buprenorphine and methadone were associated with higher treatment retention than non-pharmacotherapeutic controls and that methadone resulted in superior treatment retention to buprenorphine. At the same time, our study provides an important update to an earlier Cochrane review by Minozzi et al. (2011) [20]. This review concluded that naltrexone did not result in higher retention than placebo or non-pharmacotherapeutic agents, and that naltrexone was non-inferior to buprenorphine in treatment retention from only one study [100]. The authors’ conclusions were based on a limited number of studies that were published before June 2010. With additional evidence, we found that naltrexone is an effective treatment strategy for retention compared to non-pharmacotherapeutic interventions, which contrasts findings by Minozzi et al. (2011). Further, our NMA generated preliminary evidence on the superiority of buprenorphine to naltrexone in treatment retention, supported by both the risk ratio estimates and the average proportion of retention calculated for each medication. However, additional RCTs will need to be conducted to better ascertain the efficacy of naltrexone in relation to methadone or SROM, since the network estimates were largely based on indirect comparisons.

Evidence on naltrexone from this network meta-analysis may also have important clinical implications in relation to the current guidelines that recommend buprenorphine and methadone as first-line treatments [15–17]. A 2015 guideline by the American Society of Addiction Medicine (ASAM) described buprenorphine, methadone, and naltrexone as more effective than non-pharmacotherapeutic strategies in treating OUD but concluded that their relative advantages over each other remain unknown [18]. Our NMA partially addresses this important gap raised in the ASAM guideline by generating evidence that demonstrates greater likelihood of treatment retention associated with buprenorphine than with naltrexone. In other words, should the clinicians prioritize retention while treating patients with OUD, buprenorphine may be the preferred treatment to naltrexone. On the other hand, although methadone was ranked higher than both buprenorphine and naltrexone using the SUCRA scores, the network estimates from the methadone-naltrexone pair would need to be interpreted more cautiously. Only one trial conducted a head-to-head comparison of these two medications, which subsequently raised some concerns around heterogeneity and incoherence of the NMA results. While methadone may have higher likelihood of treatment retention than naltrexone, the extent to which this is true may require further validation through additional RCTs.

Evidence on SROM from this network meta-analysis reveals the potential methodological issues surrounding earlier trials that contributed to the current clinical practice guidelines. In Canada, for example, clinical practice guidelines have stated that SROM could be a safe and effective alternative to treating OUD based on a small number of studies comparing SROM to methadone [17, 116]. Two review studies reached similar conclusions by stating that treatment retention may be similar between SROM and methadone, but the authors of these studies also acknowledged that the methodological quality of the included studies may be low to moderate [21, 22]. Our NMA results were consistent with these reviews and guidelines, as the number of high-quality RCTs comparing SROM to other medications for OUD was limited. However, through extensive examination of the risk of bias and credibility of the NMA estimates, we also observed that most of the trials comparing SROM to methadone had been industry-sponsored by the manufacturers of SROM, namely the Mundipharma Medical Company [47, 56, 84], which had not been detected in earlier reviews. Relatedly, the recommendations in the above guidelines may be based on studies with a potential for reporting bias, further lowering the confidence in the estimates from pairwise comparisons in the NMA involving SROM. Therefore, additional RCTs comparing SROM with other pharmacotherapeutic interventions are needed to better determine comparative effectiveness and facilitate treatment decisions for opioid maintenance treatments.

Our study has several strengths. First, we conducted a rigorous and extensive literature search across five databases and grey literature. Second, we included only the trials whose participants had opioid-related disorders and whose outcomes were measured objectively, which include treatment retention and opioid use measured by urinalysis. Although this may have reduced the number of studies in the network, we applied such eligibility criteria to ensure comparable study populations across studies. Third, the network meta-analysis included all pharmacotherapeutic strategies in the model. This allowed us to derive risk ratio estimates from both direct and indirect comparisons of all treatments in the network, which was not possible in the previous pairwise meta-analyses. Fourth, we examined the credibility of our network meta-analytic estimates using the CINeMA tool, a state-of-the-art platform that enabled a thorough assessment of the NMA results across six methodological domains.

Our study also has a few limitations. First, as with all network meta-analyses, randomization holds within each study but not between different studies [117], which could lead to heterogeneity in the study population across studies and subsequently yield biased results. However, we followed a pre-specified protocol with pre-determined eligibility criteria, which allowed us to mitigate between-study population differences. Second, due to heterogeneities between studies in outcome measurement and reporting formats, we were unable to meta-analyze trials on secondary outcomes. Although we conducted NMA only on the treatment retention outcome, findings on this endpoint remain clinically relevant because improvements in retention can reduce illicit opioid use as well as psychiatric, medical, and legal setbacks, while enhancing quality of life for people with OUD [118, 119]. Third, evidence on comparisons involving naltrexone or SROM remained sparse, and confidence in the network meta-analytic estimates was low. This hindered us from drawing substantive conclusions on their efficacy and advantages in relation to the more widely prescribed buprenorphine and methadone. Fourth, about 78% of all participants across the studies in the network were male, which could compromise the generalizability of the study findings. We note, however, that the large majority of those who are treated for opioid use disorders are also male patients[13, 120–122]. Therefore, the generalizability of our study findings likely still holds true. Finally, long-term comparative effectiveness of the included pharmacotherapies remains uncertain because only RCTs were included in the network. However, the findings from our NMA serve as evidence that could pave the way for future observational studies, which are accepted as the best evidence for clinical and policy decision-making surrounding these medications [123].

## Conclusion

For treating opioid-related disorders, maintenance treatment through buprenorphine, methadone, naltrexone, and SROM is more effective than non-pharmacotherapeutic interventions. Among the medications included in the network, methadone appears to be the most efficacious pharmacotherapy for treatment retention. Due to limitations in reporting and heterogeneity in outcome measurement formats, the relative efficacy of these interventions for other clinical endpoints remains unclear. Buprenorphine and methadone appear to have superior retention to naltrexone based on a small number of studies. Upon comparison with methadone, the efficacy of slow-release oral morphine was not statistically different. However, the lack of comparison with other pharmacotherapeutic options and the potential presence of reporting bias may hinder accurate conclusions about the efficacy of SROM. Finally, our study revealed directions for future research, which include (1) further RCTs involving naltrexone or SROM to assess their relative efficacy in relation to buprenorphine and methadone, and (2) observational studies to examine long-term comparative effectiveness of these medications.

## Supporting information

S1 Text Analysis plan for using the CINeMA framework. (DOCX)

S1 Table PRISMA NMA checklist of items to include when reporting a systematic review involving a network meta-analysis of randomized controlled trials’. (DOCX)

S2 Table Search strategy. (DOCX)

S3 Table Data extraction form. (DOCX)

S4 Table List of excluded studies (full-test review) along with reasons for exclusion. (DOCX)

S5 Table Detailed description of included studies. (XLSX)

S6 Table Detailed assessment of risk of bias using RoB 2.0. (XLSX)

S7 Table Deviance information criterion (DIC) for fixed effects model versus random effects model^0^ and meta-regression analysis. ^0^ This was because the fixed effects model assumes that all studies share the same common effect, but it may not be reasonable to assume that there is one common effect size. On the other hand, the random effects model assumes that the observed estimates of the treatment effect can vary across studies due to systematic differences in the treatment effect as well as random variations due to chance [Riley, R.D., J.P. Higgins, and J.J. Deeks, Interpretation of random effects meta-analyses. BMJ, 2011. 342: p. d549.]. ^1^ Comparison with the random effects model. ^2^ Categorization of risk of bias (RoB) was based on whether the overall RoB was ‘Low risk’ or ‘Some or High risk’ using the RoB 2.0 tool. ^3^ Publication year was stratified by whether the article was published ‘Before 2010’ or ‘On or after 2010’.
(DOCX)

S8 Table Average percentage of treatment retention and Surface under the cumulative ranking (SUCRA) score for each treatment in the network. (DOCX)

S1 Fig Convergence of the Monte Carlo Markov Chain (MCMC) simulation with treatment retention as outcome. (TIF)

S2 Fig Gelman-Rubin plot to visualize convergence of the MCMC simulation with treatment retention as outcome. (TIF)

S3 Fig Rankogram of the preference of treatment options with respect to the retention outcome. (TIFF)

S4 Fig Surface under the cumulative ranking (SUCRA) score diagram for the retention outcome. (TIF)

S5 Fig Confidence in network meta-analysis (CINeMA) risk of bias contributions for each pairwise comparison based on RoB 2. (TIF)

S6 Fig Confidence in network meta-analysis (CINeMA) indirectness contributions for each pairwise comparison. (TIF)

S1 Data (CSV)
