# Supplementary material for: Relative effectiveness of medications for opioid-related disorders: A systematic review and network meta-analysis of randomized controlled trials
Source: PLoS One. 2022 Mar 31;17(3):e0266142. doi: 10.1371/journal.pone.0266142 (PMC8970369; doi:10.1371/journal.pone.0266142)
Supplement: S1 Text — (DOCX) [file pone.0266142.s001.docx]

**S1 Text. Analysis plan for using the CINeMA framework**

In the CINeMA framework, the within-study bias domain summarizes the risk of bias contributions from individual studies to the network meta-analytic estimates based on the RoB 2 tool. We assessed the presence of reporting bias by examining whether the meta-analysis was based on a small number of reported findings and whether the treatment comparison took place primarily in industry-funded trials [1]. To judge indirectness of a trial, we examined whether the study parameters, including population, interventions, outcomes, and settings of each trial, were representative of the target parameters about which we wanted to make inferences. For example, we judged that there were some concerns about indirectness if the study population consisted exclusively of male or of female patients. For imprecision, heterogeneity, and incoherence, we defined the clinically important effect size as a risk ratio of 1.50. That is, the relative effect estimates below 0.67 or above 1.50 were considered clinically important. Ratings for imprecision were algorithmically defined based on the width of the 95% confidence interval (CI), whereas the ratings for heterogeneity were algorithmically defined based on the width of the 95% prediction interval. Finally, incoherence of the network estimates was rated algorithmically based on the 95% CI of the direct and indirect RRs and the range of equivalence based on the clinically important effect size. Although we conducted our analysis using the Bayesian framework, we chose to adjudicate the quality of synthesized evidence using the CINeMA platform, which uses frequentist parameters (e.g., confidence intervals and p-values). This was because numerical estimates from a maximum likelihood model fit under the frequentist paradigm could approximate the results of a Bayesian analysis with non-informative priors [2].

REFERENCES

1. Lexchin J, Bero LA, Djulbegovic B, Clark O. Pharmaceutical industry sponsorship and research outcome and quality: systematic review. BMJ. 2003;326(7400):1167-70.

2. Jansen JP, Trikalinos T, Cappelleri JC, Daw J, Andes S, Eldessouki R, et al. Indirect treatment comparison/network meta-analysis study questionnaire to assess relevance and credibility to inform health care decision making: an ISPOR-AMCP-NPC Good Practice Task Force report. Value Health. 2014;17(2):157-73.
