# Supplementary material for: Relative effectiveness of medications for opioid-related disorders: A systematic review and network meta-analysis of randomized controlled trials
Source: PLoS One. 2022 Mar 31;17(3):e0266142. doi: 10.1371/journal.pone.0266142 (PMC8970369; doi:10.1371/journal.pone.0266142)
Supplement: S2 Table — (DOCX) [file pone.0266142.s003.docx]

**S2 Table. Search strategy**

| **Database** | **Algorithm** |
| --- | --- |
| MEDLINE | 1. exp opioid-related disorders/ 2. ((drug OR substance OR opioid* OR opiat*) adj3 (disorder* OR addict* OR abuse* OR depend*)).ti,ab. 3. 1 OR 2 4. (opioid* OR opiat* OR heroin* OR narcot*).ti,ab. 5. 3 AND 4 6. exp buprenorphine/ OR buprenorphine.ti,ab. 7. exp methadone/ OR methadone.ti,ab. 8. exp naltrexone/ OR naltrexone.ti,ab. 9. exp morphine/ OR morphine.ti,ab. 10. 6 OR 7 OR 8 OR 9 11. randomized controlled trial.pt. 12. controlled clinical trial.pt. 13. placebo.ti,ab. 14. drug therapy.sh. 15. randomly.ti,ab. 16. trial.ti,ab. 17. groups.ti,ab. 18. 11 OR 12 OR 13 OR 14 OR 15 OR 16 OR 17 19. 5 AND 10 AND 18 20. limit 19 to humans |
| EMBASE and PsycINFO | 1. exp addiction/ 2. exp drug abuse/ 3. ((drug OR substance OR opioid* OR opiat*) adj3 (disorder* OR addict* OR abuse* OR depend*)).ti,ab. 4. 1 OR 2 OR 3 5. (opioid* OR opiat* OR heroin* OR narcot*).ti,ab. 6. 4 AND 5 7. exp buprenorphine/ OR buprenorphine.ti,ab. 8. exp methadone/ OR methadone.ti,ab. 9. exp naltrexone/ OR naltrexone.ti,ab. 10. exp morphine/ OR morphine.ti,ab. 11. 7 OR 8 OR 9 OR 10 12. exp crossover procedure/ 13. exp double blind procedure/ 14. exp single blind procedure/ 15. exp controlled clinical trial/ 16. exp clinical trial/ 17. (placebo OR double blind OR single blind OR assign* OR allocat* OR volunteer*).ti,ab. 18. (random* OR factorial* OR crossover* OR (cross AND over)).ti,ab. 19. exp randomized controlled trial/ 20. 12 OR 13 OR 14 OR 15 OR 16 OR 17 OR 18 OR 19 21. 6 AND 11 AND 20 22. limit 21 to humans |
| Cochrane CENTRAL | 1. MeSH descriptor: [Opioid-Related Disorders] explode all trees 2. ((opioid* or opiat*) and (abus* or dependen* or disorder*)):ti,ab,kw 3. #1 or #2 4. "heroin":ti,ab,kw 5. (opioid* or opiat*):ti,ab,kw 6. #4 or #5 7. #3 and #6 8. MeSH descriptor: [Buprenorphine] explode all trees 9. MeSH descriptor: [Methadone] explode all trees 10. MeSH descriptor: [Naltrexone] explode all trees 11. MeSH descriptor: [Morphine] in all MeSH products 12. "buprenorphine":ti,ab,kw 13. "methadone":ti,ab,kw 14. "naltrexone":ti,ab,kw 15. "morphine":ti,ab,kw 16. #8 or #9 or #10 or #11 or #12 or #13 or #14 or #15 17. #7 and #16 (in Trials) |
| ClinicalTrials.gov | Completed Studies \| Studies With Results \| Interventional Studies \| Opioid-Related Disorders \| Buprenorphine OR methadone OR naltrexone OR morphine |
