# Supplementary material for: Relative effectiveness of medications for opioid-related disorders: A systematic review and network meta-analysis of randomized controlled trials
Source: PLoS One. 2022 Mar 31;17(3):e0266142. doi: 10.1371/journal.pone.0266142 (PMC8970369; doi:10.1371/journal.pone.0266142)
Supplement: S3 Table — (DOCX) [file pone.0266142.s004.docx]

**S3 Table. Data extraction form**

| **Variable** | **Description** |
| --- | --- |
| First_author | Last name of the first author |
| Journal | Journal where the article was published |
| Publication_year | Year in which the article was published |
| Funding | Funding source |
|  | **Study Characteristics** |
| Study_design | Study design (e.g., parallel, crossover, or factorial) |
| Randomization | Randomization (Yes/No) |
| Blinding | Blinding (e.g., single blind, double blind, or none) |
| Duration | Trial duration |
| Eligibility | Key inclusion and exclusion criteria |
| Intervention | Intervention drug/treatment |
| Comparator | Comparator drug/treatment, including active comparator, placebo, and standard of care |
| N_intervention | Number of patients randomized to the intervention group |
| N_comparator | Number of patients randomized to the comparator group |
| Trt_intervention | Treatment regimen of intervention (e.g., dose, frequency, timing, duration, and route of administration) |
| Trt_comparator | Treatment regimen of comparator (e.g., dose, frequency, timing, duration, and route of administration) |
|  | **Patient Characteristics at Baseline** |
| Mean_age | Mean age of patients |
| Male | % of male patients |
| Dz_condition | Disease condition at baseline (e.g., opioid use disorder, heroin dependence, or chronic pain with opioid dependence) |
|  | **Outcome Measures** |
| N_ret_intervention | Number of patients retained (stayed in treatment) in the intervention group |
| N_ret_comparator | Number of patients retained (stayed in treatment) in the comparator group |
| Perc_opioid_use_intervention | Percentage of urine samples in intervention group that was positive for opiates, including morphine and heroin |
| Perc_opioid_use_comparator | Percentage of urine samples in comparator group that was positive for opiates, including morphine and heroin |
| N_opioid_use_intervention | Number of patients who had at least one opiate-positive urine sample at the end of the study in the intervention group |
| N_opioid_use_comparator | Number of patients who had at least one opiate-positive urine sample at the end of the study in the control group |
