# Supplementary material for: Relative effectiveness of medications for opioid-related disorders: A systematic review and network meta-analysis of randomized controlled trials
Source: PLoS One. 2022 Mar 31;17(3):e0266142. doi: 10.1371/journal.pone.0266142 (PMC8970369; doi:10.1371/journal.pone.0266142)
Supplement: S4 Table — (DOCX) [file pone.0266142.s005.docx]

**S4 Table. List of excluded studies (full-test review) along with reasons for exclusion**

| **First Author** | **Publication Year** | **Title** | **Reasons for Exclusion** |
| --- | --- | --- | --- |
| Aalto | 2011 | Effectiveness of buprenorphine maintenance treatment as compared to a syringe exchange program among buprenorphine misusing opioid-dependent patients | Not RCT |
| Aboujaoude | 2016 | Naltrexone: A Pan-Addiction Treatment? | Not RCT |
| Abrahamsson | 2016 | Interim buprenorphine treatment in opiate dependence: A pilot effectiveness study | Not RCT |
| Abramsohn | 2009 | Sense of coherence as a stable predictor for methadone maintenance treatment (MMT) outcome | Not RCT |
| Acosta | 2011 | Buprenorphine-naloxone for the office-based treatment of opiate addiction in patients with psychiatric co-morbidities | Conference Abstract Only |
| Agar | 2001 | Buprenorphine: "field trials" of a new drug | Not RCT |
| Ahmadi | 2002 | Buprenorphine maintenance treatment of heroin dependence: The first experience from Iran | Ineligible Comparator(s) |
| Ahmadi | 2002 | A controlled trial of buprenorphine treatment for opium dependence: The first experience from Iran | Ineligible Comparator(s) |
| Ahmadi | 2002 | A randomized, clinical trial of buprenorphine maintenance treatment for Iranian patients with opioid dependency | Ineligible Comparator(s) |
| Ahmadi | 2002 | Buprenorphine treatment of opium-dependent outpatients seeking treatment in Iran | Ineligible Comparator(s) |
| Ahmadi | 2003 | Methadone versus buprenorphine maintenance for the treatment of heroin-dependent outpatients | Included |
| Ahmadi | 2003 | Controlled trial of maintenance treatment of intravenous buprenorphine dependence | Duplicate Sample |
| Ahmadi | 2003 | Controlled, randomized trial in maintenance treatment of intravenous buprenorphine dependence with naltrexone, methadone or buprenorphine: A novel study | Included |
| Ahmadi | 2003 | Twelve-month maintenance treatment of heroin-dependent outpatients with buprenorphine | Ineligible Comparator(s) |
| Ahmadi | 2003 | Treatment of intravenous buprenorphine dependence A randomized open clinical trial | Included |
| Ahmadi | 2004 | Twelve-month maintenance treatment of opium-dependent patients | Ineligible Comparator(s) |
| Ahmadi | 2004 | Treatment of heroin dependence | Duplicate Sample |
| Ahmadi | 2017 | Comparing the effect of buprenorphine and methadone in the reduction of methamphetamine craving: a randomized clinical trial | Ineligible Population |
| Ahmadi | 2018 | The effectiveness of different singly administered high doses of buprenorphine in reducing suicidal ideation in acutely depressed people with co-morbid opiate dependence: A randomized, double-blind, clinical trial | Ineligible Comparator(s) |
| Ahmadi | 2018 | Single high-dose buprenorphine for opioid craving during withdrawal | Ineligible Comparator(s) |
| Ahmadi | 2020 | Rapid effect of a single-dose buprenorphine on reduction of opioid craving and suicidal ideation: A randomized, double blind, placebo-controlled study | Ineligible Outcomes |
| Ahmed | 2019 | A scalable, automated warm handoff from the emergency department to community sites offering continued medication for opioid use disorder: Lessons learned from the EMBED trial stakeholders | Not RCT |
| Albanese | 2000 | Outcome and six month follow up of patients after Ultra Rapid Opiate Detoxification (UROD) | Not RCT |
| Amass | 2012 | A prospective, randomized, multicenter acceptability and safety study of direct buprenorphine/naloxone induction in heroin-dependent individuals | Ineligible Comparator(s) |
| Amiaz | 2008 | Naltrexone augmentation in OCD: A double-blind placebo-controlled cross-over study | Ineligible Population |
| Andorn | 2019 | 18-month safety and efficacy of RBP-6000 extended-release buprenorphine for OUD | Conference Abstract Only |
| Andorn | 2020 | Treating Opioid Use Disorder with a Monthly Subcutaneous Buprenorphine Depot Injection: 12-Month Safety, Tolerability, and Efficacy Analysis | Not RCT |
| Ang-Lee | 2006 | Single dose of 24 milligrams of buprenorphine for heroin detoxification: An open-label study of five inpatients | Not RCT |
| Anglin | 2007 | Levo-alpha-acetylmethadol (LAAM) versus methadone maintenance: 1-Year treatment retention, outcomes and status | Ineligible Comparator(s) |
| Anglin | 2009 | Longitudinal effects of LAAM and methadone maintenance on heroin addict behavior | Ineligible Comparator(s) |
| Anonymous | 1996 | Buprenorphine: A valuable alternative to methadone for long-term heroin substitute | Not RCT |
| Anonymous | 2007 | Buprenorphine + naloxone: new combination Opiate dependence: no proof of reduced risk of self-administered injection | Not RCT |
| Anonymous | 2007 | Buprenorphine + Naloxone: Opiate dependence: No proof of reduced risk of self-administered injection | Not RCT |
| Arechiga | 2019 | Naltrexone implants improve therapeutic adherence with multiple substance use disorder | Conference Abstract Only |
| Assadi | 2004 | Opioid detoxification using high doses of buprenorphine in 24 hours: A randomized, double blind, controlled clinical trial | Ineligible Comparator(s) |
| Awgu | 2010 | Heroin-dependent inmates' experiences with buprenorphine or methadone maintenance | Not RCT |
| Azatian | 1994 | A study of the use of clonidine and naltrexone in the treatment of opioid addiction in the former USSR | Not RCT |
| Badaras | 2020 | Dose escalation of naltrexone to reduce stress responses associated with opioid antagonist induction: A double-blind randomized trial | Ineligible Comparator(s) |
| Baker | 1995 | Monitoring in methadone maintenance treatment | Ineligible Comparator(s) |
| Bakhshani | 2008 | A randomized effectiveness trial of methadone, TENS and methadone plus TENS in management of opiate withdrawal symptoms | Ineligible Comparator(s) |
| Bale | 1980 | Therapeutic communities vs methadone maintenance: A prospective controlled study of narcotic addiction treatment: Design and one-year follow-up | Ineligible Outcomes |
| Balldin | 2003 | A 6-month controlled naltrexone study: Combined effect with cognitive behavioral therapy in outpatient treatment of alcohol dependence | Ineligible Population |
| Ballough | 2013 | Buprenorphine versus methadone in opioid dependant patients | Conference Abstract Only |
| Barrau | 2001 | Comparison of methadone and high dosage buprenorphine users in French care centres | Not RCT |
| Barrett | 1989 | Effects of naloxone and naltrexone on self-injury: a double-blind, placebo-controlled analysis | Ineligible Comparator(s) |
| Bearn | 1996 | Randomised double-blind comparison of lofexidine and methadone in the in-patient treatment of opiate withdrawal | Ineligible Comparator(s) |
| Beck | 2014 | Maintenance treatment for opioid dependence with slow-release oral morphine: a randomized cross-over, non-inferiority study versus methadone | Included |
| Bell | 2004 | A pilot study of buprenorphine-naloxone combination tablet (Suboxone) in treatment of opioid dependence | Not RCT |
| Bell | 2007 | A randomized trial of effectiveness and cost-effectiveness of observed versus unobserved administration of buprenorphine-naloxone for heroin dependence | Ineligible Comparator(s) |
| Bell | 2008 | Optimising the benefits of unobserved dose administration for stable opioid maintenance patients: Follow-up of a randomised trial | Not RCT |
| Bell | 2009 | The Acceptability, Safety, and Tolerability of Methadone/Naloxone in a 50: 1 Ratio | Ineligible Comparator(s) |
| Bevanda | 2017 | The differences in quality of life between the heroin addicts treated in methadone program and addicts treated in the frame of therapeutic community program | Not RCT |
| Bezie | 2004 | Compliance with methadone-based substitutive treatment: A proposed model based on immunoassay urinary sample screening | Not RCT |
| Bickel | 1987 | A clinical trial of buprenorphine: I Comparison with methadone in the detoxification of heroin addicts II Examination of its opioid blocking properties | Duplicate Sample |
| Bickel | 1988 | A clinical trial of buprenorphine: Comparison with methadone in the detoxification of heroin addicts | Included |
| Bisaga | 2018 | Outpatient transition to extended-release naltrexone in patients with opioid-use disorder | Conference Abstract Only |
| Bisaga | 2018 | Outpatient transition to extended-release injectable naltrexone for patients with opioid use disorder: A phase 3 randomized trial | Ineligible Outcomes |
| Blom | 1987 | Effects of buprenorphine in heroin addicts | Not RCT |
| Blondell | 2007 | Buprenorphine and methadone: a comparison of patient completion rates during inpatient detoxification | Not RCT |
| Blondell | 2008 | A randomized trial of extended buprenorphine detoxification for opioid dependency | Ineligible Comparator(s) |
| Blue | 2019 | Longitudinal analysis of HIV-risk behaviors of participants in a randomized trial of prison-initiated buprenorphine | Ineligible Outcomes |
| Blumberg | 2017 | Changes in quality of life in cocaine-dependent participants provided treatment with buprenorphine + naloxone & extended release naltrexone | Conference Abstract Only |
| Bond | 2012 | After the randomised injectable opiate treatment trial: Post-trial investigation of slow-release oral morphine as an alternative opiate maintenance medication | Not RCT |
| Bowen | 2017 | Mindfulness-Based Relapse Prevention for Methadone Maintenance: A Feasibility Trial | Not RCT |
| Bråbäck | 2016 | Malmö Treatment Referral and Intervention Study (MATRIS)—effective referral from syringe exchange to treatment for heroin dependence: a pilot randomized controlled trial | Ineligible Comparator(s) |
| Brahen | 1978 | Controlled clinical study of naltrexone side effects comparing first-day doses and maintenance regimens | Ineligible Comparator(s) |
| Breen | 2003 | Cessation of methadone maintenance treatment using buprenorphine: Transfer from methadone to buprenorphine and subsequent buprenorphine reductions | Ineligible Outcomes |
| Brigham | 2014 | A randomized pilot clinical trial to evaluate the efficacy of Community Reinforcement and Family Training for Treatment Retention (CRAFT-T) for improving outcomes for patients completing opioid detoxification | Ineligible Comparator(s) |
| Brinkley-Rubinstein | 2018 | A randomized, open label trial of methadone continuation versus forced withdrawal in a combined US prison and jail: Findings at 12 months post-release | Ineligible Outcomes |
| Brooks | 2010 | Long-acting injectable versus oral naltrexone maintenance therapy with psychosocial intervention for heroin dependence: A quasi-experiment | Ineligible Comparator(s) |
| Brooner | 1998 | Preliminary evidence of good treatment response in antisocial drug abusers | Ineligible Comparator(s) |
| Brooner | 2013 | Managing psychiatric comorbidity within versus outside of methadone treatment settings: a randomized and controlled evaluation | Ineligible Comparator(s) |
| Brown | 2009 | Low-dose naltrexone for disease prevention and quality of life | Not RCT |
| Budilovsky-Kelley | 2020 | Injectable weekly and monthly buprenorphine in the outpatient treatment of fentanyl users with OUD | Conference Abstract Only |
| Buntwal | 2000 | Naltrexone and lofexidine combination treatment compared with conventional lofexidine treatment for in-patient opiate detoxification | Ineligible Comparator(s) |
| Busch | 2015 | Health service use in a randomized clinical trial comparing three methods of emergency department interventions for opioid dependence | Conference Abstract Only |
| Cameron | 2006 | Pilot randomised controlled trial of community pharmacy administration of buprenorphine versus methadone | Included |
| Cami | 1990 | Double-blind assessment of buprenorphine withdrawal in opiate-addicts | Conference Abstract Only |
| Cao | 2014 | Retention and its predictors among methadone maintenance treatment clients in China: a six-year cohort study | Not RCT |
| Caplehorn | 1994 | A comparison of abstinence-oriented and indefinite methadone maintenance treatment | Not RCT |
| Caplehorn | 2004 | Prescription of heroin to treatment resistant heroin addicts: Dutch heroin trials show retention is better with methadone alone | Not RCT |
| Caplehorn | 2006 | A critical appraisal of the Australian comparative trial of methadone and buprenorphine maintenance | Duplicate Sample |
| Carrieri | 2014 | Methadone induction in primary care for opioid dependence: A pragmatic randomized trial (ANRS Methaville) | Ineligible Comparator(s) |
| Carroll | 2001 | Targeting behavioral therapies to enhance naltrexone treatment of opioid dependence: Efficacy of contingency management and significant other involvement | Ineligible Comparator(s) |
| Carroll | 2002 | Contingency management to enhance naltrexone treatment of opioid dependence: A randomized clinical trial of reinforcement magnitude | Ineligible Comparator(s) |
| Carter | 2019 | Quality of life in adult outpatients with opioid use disorder treated with long-acting subcutaneous injection buprenorphine depot: Results from an open-label multicenter clinical trial | Conference Abstract Only |
| Cavacuiti | 2003 | Managing opioid dependence Comparing buprenorphine with methadone | Not RCT |
| Chandra | 2019 | Retention in clinical trials after prison release: results from a clinical trial with incarcerated men with HIV and opioid dependence in Malaysia | Not RCT |
| Chawarski | 2008 | Behavioral drug and HIV risk reduction counseling (BDRC) with abstinence-contingent take-home buprenorphine: A pilot randomized clinical trial | Ineligible Comparator(s) |
| Chawla | 2013 | Comparison of efficacy between buprenorphine and tramadol in the detoxification of opioid (heroin)-dependent subjects | Ineligible Comparator(s) |
| Chen | 2013 | Effectiveness of prize-based contingency management in a methadone maintenance program in China | Ineligible Comparator(s) |
| Cheskin | 1994 | A controlled comparison of buprenorphine and clonidine for acute detoxification from opioids | Ineligible Comparator(s) |
| Chopra | 2009 | Buprenorphine Medication Versus Voucher Contingencies in Promoting Abstinence From Opioids and Cocaine | Ineligible Comparator(s) |
| Chou | 2016 | Comparing treatment outcomes in Veterans with opioid use disorder treated with either extended-release naltrexone or buprenorphine at Veterans Affairs Western New York (VAWNY) healthcare system | Conference Abstract Only |
| Church | 2001 | Concurrent substance use and outcome in combined behavioral and naltrexone therapy for opiate dependence | Not RCT |
| Collins | 2005 | Anesthesia-Assisted vs Buprenorphine- or Clonidine-Assisted Heroin Detoxification and Naltrexone Induction: A Randomized Trial | Ineligible Comparator(s) |
| Comer | 2001 | Buprenorphine sublingual tablets: Effects on IV heroin self-administration by humans | Ineligible Comparator(s) |
| Comer | 2002 | Intravenous buprenorphine self-administration by detoxified heroin abusers | Ineligible Outcomes |
| Comer | 2002 | Depot naltrexone: long-lasting antagonism of the effects of heroin in humans | Not RCT |
| Comer | 2005 | Comparison of intravenous buprenorphine and methadone self-administration by recently detoxified heroin-dependent individuals | Ineligible Outcomes |
| Comer | 2005 | Buprenorphine/naloxone reduces the reinforcing and subjective effects of heroin in heroin-dependent volunteers | Ineligible Comparator(s) |
| Comer | 2006 | Injectable, sustained-release naltrexone for the treatment of opioid dependence: A randomized, placebo-controlled trial | Included |
| Comer | 2020 | Transition of Patients with Opioid Use Disorder from Buprenorphine to Extended-Release Naltrexone: A Randomized Clinical Trial Assessing Two Transition Regimens | Ineligible Outcomes |
| Cornish | 1993 | Naltrexone maintenance: Effect on morphine sensitivity in normal volunteers | Not RCT |
| Cornish | 1997 | Naltrexone pharmacotherapy for opioid dependent federal probationers | Included |
| Cornish | 2012 | Six-month depot naltrexone treatment reduces relapse in parolees formerly addicted to opioids | Conference Abstract Only |
| Coviello | 2010 | A randomized trial of oral naltrexone for treating opioid-dependent offenders | Included |
| Coviello | 2012 | A multisite pilot study of extended-release injectable naltrexone treatment for previously opioid-dependent parolees and probationers | Not RCT |
| Cowan | 2005 | A randomized, double-blind, placebo-controlled, cross-over pilot study to assess the effects of long-term opioid drug consumption and subsequent abstinence in chronic noncancer pain patients receiving controlled-release morphine | Ineligible Population |
| Cozzolino | 2006 | Buprenorphine treatment: A three-year prospective study in opioid-addicted patients of a public out-patient addiction center in Milan | Not RCT |
| Cropsey | 2011 | Results of a pilot randomized controlled trial of buprenorphine for opioid dependent women in the criminal justice system | Included |
| Cropsey | 2013 | Buprenorphine and medication management in a community corrections population: A pilot Study | Not RCT |
| Cunningham | 2013 | Buprenorphine treatment outcomes among opioid-dependent cocaine users and non-users | Not RCT |
| Curran | 1999 | Additional methadone increases craving for heroin: a double-blind, placebo-controlled study of chronic opiate users receiving methadone substitution treatment | Ineligible Outcomes |
| Cushman | 2015 | Buprenorphine initiation and linkage to outpatient buprenorphine treatment does not reduce frequency of injection drug use for inpatient opioid-dependent injection drug users: Results of a randomized clinical trial | Conference Abstract Only |
| Cushman | 2016 | Buprenorphine Initiation and Linkage to Outpatient Buprenorphine do not Reduce Frequency of Injection Opiate Use Following Hospitalization | Ineligible Comparator(s) |
| Dakwar | 2015 | Naltrexone-facilitated buprenorphine discontinuation: A feasibility trial | Not RCT |
| De Jong | 2007 | High abstinence rates in heroin addicts by a new comprehensive treatment approach | Ineligible Comparator(s) |
| De Leon | 1995 | Therapeutic community methods in methadone maintenance (Passages): An open clinical trial | Not RCT |
| de los Cobos | 2000 | A controlled trial of daily versus thrice-weekly buprenorphine administration for the treatment of opiod dependence | Ineligible Comparator(s) |
| Dean | 2004 | Depressive symptoms during buprenorphine vs methadone maintenance: Findings from a randomised, controlled trial in opioid dependence | Ineligible Outcomes |
| Dean | 2006 | Does naltrexone treatment lead to depression? Findings from a randomized controlled trial in subjects with opioid dependence | Ineligible Outcomes |
| DeFulio | 2012 | Employment-based reinforcement of adherence to an FDA approved extended release formulation of naltrexone in opioid-dependent adults: A randomized controlled trial | Ineligible Comparator(s) |
| Degenhardt | 2008 | Depot naltrexone use for opioid dependence in Australia: Large-scale use of an unregistered medication in the absence of data on safety and efficacy | Not RCT |
| Demaret | 2015 | Efficacy of heroin-assisted treatment in Belgium: A randomised controlled trial | Ineligible Comparator(s) |
| Demaret | 2016 | Loss of treatment benefit when heroin-assisted treatment is stopped after 12 months | Ineligible Comparator(s) |
| Di Paola | 2014 | Design and methods of a double blind randomized placebo-controlled trial of extended-release naltrexone for HIV-infected, opioid dependent prisoners and jail detainees who are transitioning to the community | Not RCT |
| Dijkstra | 2010 | Opioid detoxification: from controlled clinical trial to clinical practice | Not RCT |
| Dolan | 2003 | A randomised controlled trial of methadone maintenance treatment versus wait list control in an Australian prison system | Ineligible Outcomes |
| Dolan | 2005 | Four-year follow-up of imprisoned male heroin users and methadone treatment: Mortality, re-incarceration and hepatitis C infection | Not RCT |
| D'Onofrio | 2015 | A randomized clinical trial of emergency department initiated treatment for opioid dependence: Two and six month outcomes | Conference Abstract Only |
| D'Onofrio | 2015 | Emergency department-initiated buprenorphine/naloxone treatment for opioid dependence: A randomized clinical trial | Included |
| D'Onofrio | 2017 | Emergency department-initiated buprenorphine for opioid dependence with continuation in primary care: outcomes during and after treatment | Conference Abstract Only |
| D'Onofrio | 2017 | Emergency Department-Initiated Buprenorphine for Opioid Dependence with Continuation in Primary Care: Outcomes During and After Intervention | Not RCT |
| Doran | 2005 | Buprenorphine induction and stabilisation in the treatment of opiate dependence | Not RCT |
| Douaihy | 2020 | Characteristics of individuals seeking to transition from BUP to XR-NTX in a randomized, placebo-controlled trial | Conference Abstract Only |
| Douaihy | 2020 | Withdrawal Patterns during Transition from Opioid Use or Buprenorphine Treatment to XR-NTX | Conference Abstract Only |
| Dunlop | 2017 | Effectiveness and cost-effectiveness of unsupervised buprenorphine-naloxone for the treatment of heroin dependence in a randomized waitlist controlled trial | Included |
| Dunn | 2015 | Employment-Based Reinforcement of Adherence to Oral Naltrexone in Unemployed Injection Drug Users: 12-Month Outcomes | Ineligible Comparator(s) |
| Dunn | 2015 | Characterizing opioid withdrawal during double-blind buprenorphine detoxification | Not RCT |
| Eaton | 2012 | Determining the clinically important difference in visual analog scale scores in abuse liability studies evaluating novel opioid formulations | Not RCT |
| Eder | 1998 | Buprenorphine and Methadone - A Comparison Trial | Conference Abstract Only |
| Eder | 1998 | Comparison of buprenorphine and methadone maintenance in opiate addicts | Duplicate Sample |
| Eder | 2002 | Delayed-release morphine and methadone for maintenance therapy in opioid dependence | Conference Abstract Only |
| Eder | 2005 | Comparative study of the effectiveness of slow-release morphine and methadone for opioid maintenance therapy | Included |
| Elarabi | 2020 | Therapeutic Drug Monitoring in Buprenorphine/Naloxone Treatment for Opioid Use Disorder: Clinical Feasibility and Optimizing Assay Precision | Not RCT |
| Elarabi | 2021 | Effectiveness of incentivised adherence and abstinence monitoring in buprenorphine maintenance: a pragmatic, randomised controlled trial | Ineligible Comparator(s) |
| Epstein | 2009 | Promoting abstinence from cocaine and heroin with a methadone dose increase and a novel contingency | Ineligible Comparator(s) |
| Escher | 2007 | Pharmacokinetic and Pharmacodynamic Properties of Buprenorphine After a Single Intravenous Administration in Healthy Volunteers: A Randomized, Double-Blind, Placebo-Controlled, Crossover Study | Ineligible Outcomes |
| Esmaeili | 2014 | Outcome evaluation of the opioid agonist maintenance treatment in Iran | Not RCT |
| Evans | 2019 | Effects of access barriers and medication acceptability on buprenorphine-naloxone treatment utilization over 2 years: results from a multisite randomized trial of adults with opioid use disorder | Ineligible Outcomes |
| Everly | 2011 | Employment-based reinforcement of adherence to depot naltrexone in unemployed opioid-dependent adults: a randomized controlled trial | Ineligible Comparator(s) |
| Falcato | 2015 | Self-reported cravings for heroin and cocaine during maintenance treatment with slow-release oral morphine compared with methadone: A randomized, crossover clinical trial | Ineligible Outcomes |
| Farabee | 2016 | Injectable pharmacotherapy for opioid use disorders (IPOD) | Not RCT |
| Farabee | 2020 | A randomized comparison of extended-release naltrexone with or without patient navigation vs enhanced treatment-as-usual for incarcerated adults with opioid use disorder | Ineligible Comparator(s) |
| Farren | 2002 | A pilot double blind placebo controlled trial of sertraline with naltrexone in the treatment of opiate dependence | Ineligible Comparator(s) |
| Fiellin | 2001 | Methadone maintenance in primary care: A randomized controlled trial | Ineligible Comparator(s) |
| Fiellin | 2002 | Treatment of heroin dependence with buprenorphine in primary care | Ineligible Comparator(s) |
| Fiellin | 2006 | Counseling plus buprenorphine-naloxone maintenance therapy for opioid dependence | Ineligible Comparator(s) |
| Fiellin | 2014 | Primary care-based buprenorphine taper vs maintenance therapy for prescription opioid dependence: A randomized clinical trial | Ineligible Comparator(s) |
| Fingerhood | 2001 | A comparison of clonidine and buprenorphine in the outpatient treatment of opiate withdrawal | Ineligible Comparator(s) |
| Fischer | 1999 | Buprenorphine versus methadone maintenance for the treatment of opioid dependence | Included |
| Fischer | 1999 | Buprenorphine vs methadone as maintenance treatment for opioid dependence | Duplicate Sample |
| Foltin | 1996 | Effects of methadone or buprenorphine maintenance on the subjective and reinforcing effects of intravenous cocaine in humans | Ineligible Outcomes |
| Foy | 1998 | An open trial of naltrexone for opiate dependence | Not RCT |
| Friedman | 1994 | Retention of patients who entered methadone maintenance via an interim methadone clinic | Not RCT |
| Friedmann | 2015 | Patient selection for extended-release naltrexone among criminal justice-involved persons with opioid use disorder | Conference Abstract Only |
| Friedmann | 2018 | Initiation of extended release naltrexone (XR-NTX) for opioid use disorder prior to release from prison | Ineligible Comparator(s) |
| Fudala | 1998 | Effects of buprenorphine and naloxone in morphine-stabilized opioid addicts | Ineligible Outcomes |
| Galanter | 2003 | Short-Term Buprenorphine Maintenance: Treatment Outcome | Not RCT |
| Gerra | 2004 | Buprenorphine versus methadone for opioid dependence: Predictor variables for treatment outcome | Not RCT |
| Giacomuzzi | 2006 | Opioid addicts at admission vs slow-release oral morphine, methadone, and sublingual buprenorphine maintenance treatment participants | Ineligible Outcomes |
| Gibson | 2003 | A comparison of buprenorphine treatment in clinic and primary care settings: A randomised trial | Not RCT |
| Gordon | 2008 | A randomized clinical trial of methadone maintenance for prisoners: Findings at 6 months post-release | Duplicate Sample |
| Gordon | 2014 | A randomized controlled trial of prison-initiated buprenorphine: Prison outcomes and community treatment entry | Duplicate Sample |
| Gordon | 2017 | A randomized clinical trial of buprenorphine for prisoners: Findings at 12-months post-release | Ineligible Comparator(s) |
| Gordon | 2017 | Extended-release naltrexone for pre-release prisoners: A randomized trial of medical mobile treatment | Not RCT |
| Gordon | 2019 | A randomized controlled trial of buprenorphine for probationers and parolees: Bridging the gap into treatment | Not RCT |
| Greiner | 2021 | Naturalistic follow-up after a trial of medications for opioid use disorder: Medication status, opioid use, and relapse | Not RCT |
| Gross | 2006 | A comparison between low-magnitude voucher and buprenorphine medication contingencies in promoting abstinence from opioids and cocaine | Ineligible Comparator(s) |
| Gruber | 2008 | A randomized trial of 6-month methadone maintenance with standard or minimal counseling versus 21-day methadone detoxification | Included |
| Gryczynski | 2013 | Retention in methadone and buprenorphine treatment among African Americans | Not RCT |
| Gunne | 1981 | The Swedish methadone maintenance program: A controlled study | Ineligible Outcomes |
| Guo | 2001 | Efficacy of naltrexone hydrochloride for preventing relapse among opiate-dependent patients after detoxification | Included |
| Haasen | 2007 | Heroin-assisted treatment for opioid dependence: randomised controlled trial | Ineligible Comparator(s) |
| Haasen | 2010 | Is heroin-assisted treatment effective for patients with no previous maintenance treatment? results from a German randomised controlled trial | Ineligible Comparator(s) |
| Haeny | 2020 | Extended-release naltrexone versus buprenorphine-naloxone to treat opioid use disorder among black adults | Not RCT |
| Haight | 2019 | Efficacy and safety of a monthly buprenorphine depot injection for opioid use disorder: a multicentre, randomised, double-blind, placebo-controlled, phase 3 trial | Included |
| Hammig | 2014 | Safety and tolerability of slow-release oral morphine versus methadone in the treatment of opioid dependence | Duplicate Sample |
| Hoffman | 2017 | Safety of a Rapidly Dissolving Buprenorphine/Naloxone Sublingual Tablet (BNX-RDT) for Treatment of Opioid Dependence: A Multicenter, Open-label Extension Study | Not RCT |
| Hollister | 1978 | Clinical evaluation of naltrexone treatment of opiate-dependent individuals | Included |
| Howells | 2002 | Prison based detoxification for opioid dependence: A randomised double blind controlled trial of lofexidine and methadone | Ineligible Comparator(s) |
| Hser | 2014 | Treatment retention among patients randomized to buprenorphine/naloxone compared to methadone in a multi-site trial | Duplicate Sample |
| Hser | 2016 | Long-term outcomes after randomization to buprenorphine/naloxone versus methadone in a multi-site trial | Not RCT |
| Hser | 2021 | Long-term follow-up assessment of opioid use outcomes among individuals with comorbid mental disorders and opioid use disorder treated with buprenorphine or methadone in a randomized clinical trial | Duplicate Sample |
| Jarvis | 2019 | The effects of extended-release injectable naltrexone and incentives for opiate abstinence in heroin-dependent adults in a model therapeutic workplace: A randomized trial | Included |
| Jayaram-Lindstrom | 2008 | Naltrexone attenuates the subjective effects of amphetamine in patients with amphetamine dependence | Ineligible Population |
| Jayaram-Lindström | 2004 | Effects of naltrexone on the subjective response to amphetamine in healthy volunteers | Ineligible Population |
| Jittiwutikarn | 2004 | Comparison of tincture of opium and methadone to control opioid withdrawal in a Thai treatment centre | Not RCT |
| Johnson | 1992 | A controlled trial of buprenorphine treatment for opioid dependence | Included |
| Johnson | 1995 | A placebo controlled clinical trial of buprenorphine as a treatment for opioid dependence | Included |
| Johnson | 2000 | A comparison of levomethadyl acetate, buprenorphine, and methadone for opioid dependence | Included |
| Kakko | 2003 | 1-year retention and social function after buprenorphine-assisted relapse prevention treatment for heroin dependence in Sweden: A randomised, placebo-controlled trial | Included |
| Kakko | 2007 | A stepped care strategy using buprenorphine and methadone versus conventional methadone maintenance in heroin dependence: A randomized controlled trial | Included |
| Kamien | 2008 | Buprenorphine-naloxone versus methadone maintenance therapy: A randomised double-blind trial with opioid-dependent patients | Included |
| Kastelic | 2008 | Slow-release oral morphine for maintenance treatment of opioid addicts intolerant to methadone or with inadequate withdrawal suppression | Not RCT |
| Kelly | 2020 | Impact of methadone treatment initiated in jail on subsequent arrest | Ineligible Outcomes |
| Khalili | 2019 | A double blind randomized clinical trial of buprenorphine augmentation for treatment of psychotic symptoms in opioid addicted bipolar patients | Ineligible Outcomes |
| King | 2002 | A multicenter randomized evaluation of methadone medical maintenance | Ineligible Comparator(s) |
| King | 2006 | A 12-month controlled trial of methadone medical maintenance integrated into an adaptive treatment model | Ineligible Comparator(s) |
| Kinlock | 2007 | A randomized clinical trial of methadone maintenance for prisoners: Results at 1-month post-release | Duplicate Sample |
| Kinlock | 2009 | A randomized clinical trial of methadone maintenance for prisoners: Results at 12 months postrelease | Included |
| Kirtadze | 2012 | Behavioral treatment + naltrexone reduces drug use and legal problems in the Republic of Georgia | Ineligible Outcomes |
| Kornor | 2006 | Abstinence-orientated buprenorphine replacement therapy for young adults in out-patient counselling | Not RCT |
| Korthuis | 2017 | Feasibility and safety of extended-release naltrexone treatment of opioid and alcohol use disorder in HIV clinics: a pilot/feasibility randomized trial | Ineligible Population |
| Korthuis | 2017 | Extended-release naltrexone feasibility in HIV clinics: a pilot study | Conference Abstract Only |
| Korthuis | 2021 | HIV clinic-based buprenorphine plus naloxone versus referral for methadone maintenance therapy for treatment of opioid use disorder in HIV clinics in Vietnam (BRAVO): an open-label, randomised, non-inferiority trial | Included |
| Kosten | 1988 | Buprenorphine detoxification from opioid dependence: A pilot study | Ineligible Comparator(s) |
| Kosten | 1993 | Buprenorphine versus methadone maintenance for opioid dependence | Included |
| Kristensen | 2005 | Buprenorphine and methadone to opiate addicts--a randomized trial | Included |
| Krook | 2002 | A placebo-controlled study of high dose buprenorphine in opiate dependents waiting for medication-assisted rehabilitation in Oslo, Norway | Included |
| Krupitsky | 2004 | Naltrexone for heroin dependence treatment in St Petersburg, Russia | Included |
| Krupitsky | 2011 | Injectable extended-release naltrexone for opioid dependence: A double-blind, placebo-controlled, multicentre randomised trial | Included |
| Krupitsky | 2012 | Randomized trial of long-acting sustained-release naltrexone implant vs oral naltrexone or placebo for preventing relapse to opioid dependence | Included |
| Krupitsky | 2013 | Injectable extended-release naltrexone (XR-NTX) for opioid dependence: long-term safety and effectiveness | Not RCT |
| Krupitsky | 2014 | Injectable extended-release naltrexone for opioid dependence: an open label study of long-term safety and efficacy | Not RCT |
| Krupitsky | 2015 | A double-blind randomized placebo-controlled study of the efficacy of the combined treatment with naltrexone and guanfacine for relapse prevention in opiate dependence | Duplicate Sample |
| Kunoe | 2009 | Naltrexone implants after in-patient treatment for opioid dependence: Randomised controlled trial | Ineligible Outcomes |
| Lange | 1990 | Safety and side-effects of buprenorphine in the clinical management of heroin addiction | Ineligible Comparator(s) |
| Latif | 2019 | Anxiety, Depression, and Insomnia among Adults with Opioid Dependence Treated with Extended-Release Naltrexone vs Buprenorphine-Naloxone: A Randomized Clinical Trial and Follow-up Study | Duplicate Sample |
| Law | 2017 | Buprenorphine/naloxone versus methadone and lofexidine in community stabilisation and detoxification: A randomised controlled trial of low dose short-term opiate-dependent individuals | Ineligible Comparator(s) |
| Lee | 2015 | Opioid treatment at release from jail using extended-release naltrexone: a pilot proof-of-concept randomized effectiveness trial | Included |
| Lee | 2016 | Extended-release naltrexone to prevent opioid relapse in criminal justice offenders | Included |
| Lee | 2018 | Comparative effectiveness of extended-release naltrexone versus buprenorphine-naloxone for opioid relapse prevention (X:BOT): a multicentre, open-label, randomised controlled trial | Included |
| Lerner | 1992 | A Naltrexone double blind placebo controlled study in Israel | Included |
| Leslie | 2015 | Effects of injectable extended-release naltrexone (XR-NTX) for opioid dependence on residential rehabilitation outcomes and early follow-up | Not RCT |
| Liebschutz | 2014 | Buprenorphine treatment for hospitalized, opioid-dependent patients: A randomized clinical trial | Ineligible Comparator(s) |
| Liebschutz | 2017 | Improving adherence to long-term opioid therapy guidelines to reduce opioid misuse in primary care: A cluster-randomized clinical trial | Ineligible Comparator(s) |
| Ling | 1996 | A controlled trial comparing buprenorphine and methadone maintenance in opioid dependence | Included |
| Ling | 2010 | Buprenorphine implants for treatment of opioid dependence: A randomized controlled trial | Included |
| Ling | 2013 | Comparison of behavioral treatment conditions in buprenorphine maintenance | Ineligible Comparator(s) |
| Lintzeris | 2002 | A randomized controlled trial of buprenorphine in the management of short-term ambulatory heroin withdrawal | Ineligible Comparator(s) |
| Lobmaier | 2010 | Naltrexone implants compared to methadone: Outcomes six months after prison release | Ineligible Outcomes |
| Lofwall | 2005 | Comparative safety and side effect profiles of buprenorphine and methadone in the outpatient treatment of opioid dependence | Ineligible Outcomes |
| Lopatko | 2003 | Opioid effects and opioid withdrawal during a 24 h dosing interval in patients maintained on buprenorphine | Ineligible Comparator(s) |
| Lucas | 2010 | Clinic-based treatment of opioid-dependent HIV-infected patients versus referral to an opioid treatment program: A randomized trial | Ineligible Comparator(s) |
| Madlung-Kratzer | 2009 | A double-blind, randomized, parallel group study to compare the efficacy, safety and tolerability of slow-release oral morphine versus methadone in opioid-dependent in-patients willing to undergo detoxification | Included |
| Magura | 2009 | Buprenorphine and methadone maintenance in jail and post-release: A randomized clinical trial | Included |
| Mannelli | 2007 | Effectiveness of low-dose naltrexone in the post-detoxification treatment of opioid dependence | Not RCT |
| Mannelli | 2009 | Very low dose naltrexone addition in opioid detoxification: A randomized, controlled trial | Ineligible Comparator(s) |
| Mannelli | 2009 | Early outcomes following low dose naltrexone enhancement of opioid detoxification | Not RCT |
| Mannelli | 2014 | Extended release naltrexone injection is performed in the majority of opioid dependent patients receiving outpatient induction: A very low dose naltrexone and buprenorphine open label trial | Not RCT |
| Mattick | 2003 | Buprenorphine versus methadone maintenance therapy: A randomized double-blind trial with 405 opioid-dependent patients | Included |
| McDermott | 2015 | Initial response as a predictor of 12-week buprenorphine-naloxone treatment response in a prescription opioid-dependent population | Not RCT |
| McKenzie | 2012 | A randomized trial of methadone initiation prior to release from incarceration | Ineligible Comparator(s) |
| Mello | 1981 | Operant analysis of human heroin self-administration and the effects of naltrexone | Ineligible Outcomes |
| Mello | 1982 | Buprenorphine effects on human heroin self-administration: An operant analysis | Ineligible Outcomes |
| Mendelson | 1996 | Buprenorphine and naloxone interactions in opiate-dependent volunteers | Ineligible Outcomes |
| Mendelson | 1999 | Buprenorphine and naloxone combinations: The effects of three dose ratios in morphine-stabilized, opiate-dependent volunteers | Ineligible Outcomes |
| Mitchell | 2004 | Slow-release oral morphine versus methadone: A crossover comparison of patient outcomes and acceptability as maintenance pharmacotherapies for opioid dependence | Ineligible Outcomes |
| Mitchell | 2015 | Changes in Quality of Life following Buprenorphine Treatment: Relationship with Treatment Retention and Illicit Opioid Use | Not RCT |
| Mitchell | 2021 | HIV-Risk Behavior Among Adults with Opioid Use Disorder During 12 Months Following Pre-trial Detention: results from a Randomized Trial of Methadone Treatment | Ineligible Comparator(s) |
| Mitchell | 2021 | An alternative analysis of illicit opioid use during treatment in a randomized trial of extended-release naltrexone versus buprenorphine-naloxone: A per-protocol and completers analysis | Duplicate Sample |
| Mokri | 2016 | Medical treatments for opioid use disorder in Iran: a randomized, double-blind placebo-controlled comparison of buprenorphine/naloxone and naltrexone maintenance treatment | Included |
| Monico | 2018 | Treatment outcomes among a cohort of African American buprenorphine patients: Follow-up at 12 months | Not RCT |
| Montoya | 2004 | Randomized trial of buprenorphine for treatment of concurrent opiate and cocaine dependence | Ineligible Comparator(s) |
| Nasser | 2016 | Sustained-release buprenorphine (RBP-6000) blocks the effects of opioid challenge with hydromorphone in subjects with opioid use disorder | Ineligible Outcomes |
| Neri | 2005 | Randomized clinical trial to compare the effects of methadone and buprenorphine on the immune system in drug abusers | Included |
| Neumann | 2013 | A preliminary study comparing methadone and buprenorphine in patients with chronic pain and coexistent opioid addiction | Included |
| Neumann | 2020 | Randomized clinical trial comparing buprenorphine/naloxone and methadone for the treatment of patients with failed back surgery syndrome and opioid addiction | Included |
| Newman | 1979 | Double-blind comparison of methadone and placebo maintenance treatments of narcotic addicts in Hong Kong | Included |
| Nunes | 2015 | Treating opioid dependence with injectable extended-release naltrexone (XR-NTX): Who will respond? | Duplicate Sample |
| Nunes | 2018 | Effects of extended-release naltrexone extend beyond exogenous opioid blockade: clinical trial observations | Conference Abstract Only |
| Nunes | 2018 | Relapse to opioid use disorder after inpatient treatment: Protective effect of injection naltrexone | Duplicate Sample |
| Nunes | 2020 | Opioid use and dropout from extended-release naltrexone in a controlled trial: implications for mechanism | Duplicate Sample |
| Ochoa | 1992 | Treatment with naltrexone in opiate dependents: 2 years' follow-up [Spanish] | Not RCT |
| O'Connor | 1995 | Primary care-based ambulatory opioid detoxification: The results of a clinical trial | Not RCT |
| O'Connor | 1996 | A pilot study of primary-care-based buprenorphine maintenance for heroin dependence | Not RCT |
| O'Connor | 1998 | A randomized trial of buprenorphine maintenance for heroin dependence in a primary care clinic for substance users versus a methadone clinic | Ineligible Comparator(s) |
| Oldham | 2004 | The Leeds Evaluation of Efficacy of Detoxification Study (LEEDS) project: an open-label pragmatic randomised control trial comparing the efficacy of differing therapeutic agents for primary care detoxification from either street heroin or methadone | Not RCT |
| Oliveto | 1994 | Cocaine use in buprenorphine- vs methadone-maintained patients | Duplicate Sample |
| Oliveto | 1999 | Desipramine in opioid-dependent cocaine abusers maintained on buprenorphine vs methadone | Included |
| Opheim | 2021 | Risk of Relapse Among Opioid‐Dependent Patients Treated With Extended‐Release Naltrexone or Buprenorphine‐Naloxone: A Randomized Clinical Trial | Ineligible Outcomes |
| Otiashvili | 2012 | Drug use and HIV risk outcomes in opioid-injecting men in the Republic of Georgia: Behavioral treatment+naltrexone compared to usual care | Included |
| Otiashvili | 2013 | Methadone and buprenorphine-naloxone are effective in reducing illicit buprenorphine and other opioid use, and reducing HIV risk behavior--outcomes of a randomized trial | Included |
| Pani | 2000 | Buprenorphine: A controlled clinical trial in the treatment of opioid dependence | Included |
| Petitjean | 2001 | Double-blind randomized trial of buprenorphine and methadone in opiate dependence | Included |
| Pinto | 2008 | A pilot study for a randomized controlled and patient preference trial of buprenorphine versus methadone maintenance treatment in the management of opiate dependent patients | Not RCT |
| Pinto | 2010 | The SUMMIT Trial: A field comparison of buprenorphine versus methadone maintenance treatment | Not RCT |
| Piralishvili | 2015 | Opioid Addicted Buprenorphine Injectors: Drug Use During and After 12-Weeks of Buprenorphine-Naloxone or Methadone in the Republic of Georgia | Duplicate Sample |
| Preston | 2000 | Methadone dose increase and abstinence reinforcement for treatment of continued heroin use during methadone maintenance | Ineligible Comparator(s) |
| Preston | 2002 | Abstinence reinforcement maintenance contingency and one-year follow-up | Ineligible Comparator(s) |
| Rabinowitz | 1997 | Compliance to naltrexone treatment after ultra-rapid opiate detoxification: An open label naturalistic study | Not RCT |
| Rabinowitz | 2002 | Outcomes of naltrexone maintenance following ultra rapid opiate detoxification versus intensive inpatient detoxification | Not RCT |
| Raistrick | 2005 | A comparison of buprenorphine and lofexidine for community opiate detoxification: Results from a randomized controlled trial | Ineligible Comparator(s) |
| Raleigh | 2017 | Buprenorphine maintenance vs Placebo for opioid dependence | Not RCT |
| Rapeli | 2007 | Methadone vs buprenorphine/naloxone during early opioid substitution treatment: a naturalistic comparison of cognitive performance relative to healthy controls | Not RCT |
| Rashid | 2012 | Efficacy of naltrexone in preventing relapse in opioid dependent patients | Ineligible Comparator(s) |
| Rea | 2004 | A randomised, controlled trial of low dose naltrexone for the treatment of opioid dependence | Ineligible Comparator(s) |
| Reimer | 2011 | Physical and mental health in severe opioid-dependent patients within a randomized controlled maintenance treatment trial | Ineligible Comparator(s) |
| Renzelli | 2006 | Less pain, more gain: Buprenorphine-naloxone and patient retention in treatment | Not RCT |
| Resnick | 1992 | Buprenorphine: An alternative to methadone for heroin dependence treatment | Ineligible Comparator(s) |
| Resnick | 2001 | Buprenorphine treatment of heroin dependence (detoxification and maintenance) in a private practice setting | Not RCT |
| Rhoades | 1998 | Retention, HIV risk, and illicit drug use during treatment: Methadone dose and visit frequency | Ineligible Comparator(s) |
| Rich | 2015 | Methadone continuation versus forced withdrawal on incarceration in a combined US prison and jail: a randomised, open-label trial | Ineligible Comparator(s) |
| Ritter | 2003 | A randomized trial comparing levo-alpha acetylmethadol with methadone maintenance for patients in primary care settings in Australia | Ineligible Comparator(s) |
| Robertson | 2006 | Addressing the efficacy of dihydrocodeine versus methadone as an alternative maintenance treatment for opiate dependence: A randomized controlled trial | Ineligible Comparator(s) |
| Roche | 2017 | Naltrexone moderates the relationship between cue-induced craving and subjective response to methamphetamine in individuals with methamphetamine use disorder | Ineligible Population |
| Rosado | 2007 | Sublingual buprenorphine/naloxone precipitated withdrawal in subjects maintained on 100 mg of daily methadone | Ineligible Outcomes |
| Rosenthal | 2013 | Buprenorphine implants for treatment of opioid dependence: randomized comparison to placebo and sublingual buprenorphine/naloxone | Included |
| Rosenthal | 2016 | Effect of buprenorphine implants on illicit opioid use among abstinent adults with opioid dependence treated with sublingual buprenorphine a randomized clinical trial | Ineligible Comparator(s) |
| Rosenthal | 2017 | Sensitivity analysis of a comparative trial of 6 month buprenorphine implants (probuphine) and sublingual buprenorphine in stable opioid-dependent patients | Ineligible Comparator(s) |
| Rosenthal | 2017 | A randomized trial of buprenorphine implants in adults stabilized on sublingual buprenorphine | Conference Abstract Only |
| Rothenberg | 2002 | Behavioral naltrexone therapy: an integrated treatment for opiate dependence | Not RCT |
| Rudolph | 2021 | Optimizing opioid use disorder treatment with naltrexone or buprenorphine | Duplicate Sample |
| Ruglass | 2019 | Trajectory classes of opioid use among individuals in a randomized controlled trial comparing extended-release naltrexone and buprenorphine-naloxone | Not RCT |
| Runarsdottir | 2017 | Extended-Release Injectable Naltrexone (XR-NTX) with Intensive Psychosocial Therapy for Amphetamine-Dependent Persons Seeking Treatment: A Placebo-Controlled Trial | Ineligible Population |
| Salehi | 2015 | The Effect of Buprenorphine on Methamphetamine Cravings | Ineligible Population |
| Salisbury-Afshar | 2015 | Buprenorphine maintenance vs methadone maintenance or placebo for opioid use disorder | Not RCT |
| Sam | 1990 | Double blind assessment of buprenorphine withdrawal in opiate-addicts | Conference Abstract Only |
| San | 1990 | Efficacy of clonidine, guanfacine and methadone in the rapid detoxification of heroin addicts: a controlled clinical trial | Ineligible Comparator(s) |
| San | 1991 | Follow-up after a six-month maintenance period on naltrexone versus placebo in heroin addicts | Included |
| Saunders | 2002 | Comparison of rapid opiate detoxification and naltrexone with methadone maintenance in the treatment of opiate dependence: a randomized controlled trial | Conference Abstract Only |
| Saxon | 2013 | Buprenorphine/Naloxone and methadone effects on laboratory indices of liver health: A randomized trial | Included |
| Schottenfeld | 1997 | Buprenorphine vs methadone maintenance treatment for concurrent opioid dependence and cocaine abuse | Included |
| Schottenfeld | 2005 | Methadone versus buprenorphine with contingency management or performance feedback for cocaine and opioid dependence | Included |
| Schottenfeld | 2008 | Maintenance treatment with buprenorphine and naltrexone for heroin dependence in Malaysia: a randomised, double-blind, placebo-controlled trial | Included |
| Schottenfeld | 2021 | Behavioral counseling and abstinence-contingent take-home buprenorphine in general practitioners’ offices in Malaysia: a randomized, open-label clinical trial | Included |
| Schwartz | 2006 | A randomized controlled trial of interim methadone maintenance | Included |
| Schwartz | 2007 | A randomized controlled trial of interim methadone maintenance: 10-Month follow-up | Ineligible Comparator(s) |
| Schwartz | 2017 | Patient-centered methadone treatment: a randomized clinical trial | Ineligible Comparator(s) |
| Schwartz | 2020 | Methadone treatment of arrestees: A randomized clinical trial | Included |
| Schwartz | 2021 | Randomized trial of methadone treatment of arrestees: 24-month post-release outcomes | Duplicate Sample |
| Sees | 2000 | Methadone maintenance vs 180-day psychosocially enriched detoxification for treatment of opioid dependence: A randomized controlled trial | Ineligible Comparator(s) |
| Seifert | 2002 | Detoxification of opiate addicts with multiple drug abuse: A comparison of buprenorphine vs methadone | Included |
| Setnik | 2013 | A clinical trial to determine if corelease of morphine and naltrexone from crushed extended-release capsules induces withdrawal in opioid-dependent patients: A descriptive analysis of six patients | Ineligible Population |
| Sheard | 2009 | The Leeds Evaluation of Efficacy of Detoxification Study (LEEDS) prisons project: a randomised controlled trial comparing dihydrocodeine and buprenorphine for opiate detoxification | Ineligible Comparator(s) |
| Shufman | 1994 | The efficacy of Naltrexone in preventing reabuse of heroin after detoxification | Included |
| Sigmon | 2004 | Evaluation of an injection depot formulation of buprenorphine: Placebo comparison | Ineligible Outcomes |
| Sigmon | 2009 | Brief buprenorphine detoxification for the treatment of prescription opioid dependence: A pilot study | Not RCT |
| Sigmon | 2013 | A randomized, double-blind evaluation of buprenorphine taper duration in primary prescription opioid abusers | Ineligible Comparator(s) |
| Sobel | 2004 | Open-label trial of an injection depot formulation of buprenorphine in opioid detoxification | Ineligible Outcomes |
| Sofuoglu | 2003 | Effects of naltrexone and isradipine, alone or in combination, on cocaine responses in humans | Ineligible Outcomes |
| Solli | 2019 | Availability of extended-release naltrexone may increase the number of opioid-dependent individuals in treatment: Extension of a randomized clinical trial | Not RCT |
| Soyka | 2008 | Retention rate and substance use in methadone and buprenorphine maintenance therapy and predictors of outcome: Results from a randomized study | Included |
| Srivastava | 2019 | Buprenorphine in the emergency department: randomized clinical controlled trial of clonidine versus buprenorphine for the treatment of opioid withdrawal | Ineligible Comparator(s) |
| Stauffer | 2009 | Subjective effects and safety of whole and tampered morphine sulfate and naltrexone hydrochloride (ALO-01) extended-release capsules versus morphine solution and placebo in experienced non-dependent opioid users: A randomized, double-blind, placebo-controlled, crossover study | Ineligible Population |
| Stein | 2020 | Initiating buprenorphine treatment for opioid use disorder during short-term in-patient 'detoxification': a randomized clinical trial | Ineligible Comparator(s) |
| Stine | 1994 | Reduction of opiate withdrawal-like symptoms by cocaine abuse during methadone and buprenorphine maintenance | Ineligible Outcomes |
| Stoller | 2001 | Effects of buprenorphine/naloxone in opioid-dependent humans | Ineligible Outcomes |
| Strain | 1994 | Buprenorphine versus methadone in the treatment of opioid-dependent cocaine users | Included |
| Strain | 1994 | Comparison of buprenorphine and methadone in the treatment of opioid dependence | Included |
| Strain | 1996 | Buprenorphine versus methadone in the treatment of opioid dependence: Self-Reports, urinalysis, and addiction severity index | Duplicate Sample |
| Strang | 2019 | Extended-release naltrexone versus standard oral naltrexone versus placebo for opioid use disorder: The NEAT three-arm RCT | Not RCT |
| Sullivan | 2006 | A trial of integrated buprenorphine/naloxone and HIV clinical care | Ineligible Comparator(s) |
| Sullivan | 2013 | Naltrexone treatment for opioid dependence: Does its effectiveness depend on testing the blockade? | Duplicate Sample |
| Sullivan | 2015 | Long-acting injectable naltrexone induction: A randomized trial of outpatient opioid detoxification with naltrexone vs Buprenorphine | Conference Abstract Only |
| Sullivan | 2015 | Opioid use and dropout in patients receiving oral naltrexone with or without single administration of injection naltrexone | Included |
| Sullivan | 2017 | Long-acting injectable naltrexone induction: A randomized trial of outpatient opioid detoxification with naltrexone versus buprenorphine | Ineligible Outcomes |
| Sullivan | 2019 | A randomized trial comparing extended-release injectable suspension and oral naltrexone, both combined with behavioral therapy, for the treatment of opioid use disorder | Ineligible Comparator(s) |
| Tabassomi | 2016 | Opium tincture versus methadone syrup in management of acute raw opium withdrawal: A randomized, double-blind, controlled trial | Ineligible Outcomes |
| Tan | 2017 | Opioid medication use in the surgical patient: An assessment of prescribing patterns and utilization | Conference Abstract Only |
| Tanum | 2016 | Optimal prevention of relapse among opioid users: A 12-week randomized controlled trial of extended-release naltrexone injections versus daily buprenorphine-naloxone | Conference Abstract Only |
| Tanum | 2017 | Effectiveness of injectable extended-release naltrexone vs daily buprenorphine-naloxone for opioid dependence: A randomized clinical noninferiority trial | Included |
| Tanum | 2017 | Optimal prevention of relapse among opioid users: a 12-week randomized controlled trial of extended-release naltrexone injections versus daily buprenorphine-naloxone | Conference Abstract Only |
| Throckmorton | 2011 | Injectable extended-release naltrexone for opioid dependence | Not RCT |
| Tiihonen | 2012 | Naltrexone implant for the treatment of polydrug dependence: A randomized controlled trial | Included |
| Tompkin | 2019 | Daily withdrawal and craving scores as predictors of dropout during outpatient induction onto XR-NTX | Conference Abstract Only |
| Tompkins | 2014 | A double blind, within subject comparison of spontaneous opioid withdrawal from buprenorphine versus morphine | Ineligible Outcomes |
| Tucker | 2004 | Naltrexone maintenance for heroin dependence: Uptake, attrition and retention | Not RCT |
| U.S. National Research Council | 1978 | Clinical evaluation of naltrexone treatment of opiate-dependent individuals Report of the National Research Council Committee on Clinical Evaluation of Narcotic Antagonists | Duplicate Sample |
| Uehlinger | 1998 | Comparison of buprenorphine and methadone in the treatment of opioid dependence Swiss multicentre study | Duplicate Sample |
| Umbricht | 1999 | Naltrexone shortened opioid detoxification with buprenorphine | Ineligible Comparator(s) |
| Umbricht | 2003 | Opioid detoxification with buprenorphine, clonidine, or methadone in hospitalized heroin-dependent patients with HIV infection | Ineligible Outcomes |
| Umbricht | 2004 | Effects of high-dose intravenous buprenorphine in experienced opioid abusers | Ineligible Outcomes |
| Valerie Curran | 1999 | Additional methadone increases craving for heroin: A double blind, placebo-controlled study of chronic opiate users receiving methadone substitution treatment | Ineligible Outcomes |
| Vanicheseni | 1991 | A controlled trial of methadone maintenance in a population of intravenous drug users in Bangkok: Implications for prevention of HIV | Ineligible Comparator(s) |
| Vasilev | 2006 | Safety and efficacy of oral slow release morphine for maintenance treatment in heroin addicts: A 6-month open noncomparative study | Not RCT |
| Verrando | 2005 | Methadone and buprenorphine maintenance therapies for patients with hepatitis C virus infected after intravenous drug use | Not RCT |
| Verthein | 2004 | Treatment of opiate addicts with buprenorphine: A prospective naturalistic trial | Not RCT |
| Verthein | 2015 | Mental symptoms and drug use in maintenance treatment with slow-release oral morphine compared to methadone: Results of a randomized crossover study | Duplicate Sample |
| Vidjak | 2003 | Treating heroin addiction: Comparison of methadone therapy, hospital therapy without methadone, and therapeutic community | Not RCT |
| Vilalta | 1987 | Methadone, clonidine and levomepromazine in the treatment of opiate abstinence syndrome: double-blind clinical trial in heroin-addicted patients admitted to a general hospital for organic pathology [Spanish] | Ineligible Population |
| Villano | 2002 | Improving treatment engagement and outcomes for cocaine-using methadone patients | Ineligible Comparator(s) |
| Vocci | 2015 | Buprenorphine dose induction in non-opioid-tolerant pre-release prisoners | Not RCT |
| Vocci | 2016 | A randomized trial of probuphine® implants in adults stabilized on sublingual buprenorphine | Conference Abstract Only |
| Waal | 2006 | Naltrexone implants -- duration, tolerability and clinical usefulness A pilot study | Not RCT |
| Waddell | 2021 | Long-acting buprenorphine vs naltrexone opioid treatments in CJS-involved adults (EXIT-CJS) | Not RCT |
| Walsh | 1993 | Comparison of the acute effects of buprenorphine and methadone in non- dependent humans | Conference Abstract Only |
| Walsh | 1995 | Effects of buprenorphine and methadone in methadone-maintained subjects | Ineligible Outcomes |
| Walsh | 2003 | Evaluation of the effects of lofexidine and clonidine on naloxone-precipitated withdrawal in opioid-dependent humans | Ineligible Comparator(s) |
| Walsh | 2015 | Intranasal buprenorphine alone and in combination with naloxone: Reinforcing efficacy and abuse liability in physically dependent opioid abusers | Conference Abstract Only |
| Walsh | 2016 | Intranasal buprenorphine alone and in combination with naloxone: Abuse liability and reinforcing efficacy in physically dependent opioid abusers | Ineligible Outcomes |
| Walsh | 2017 | Effect of buprenorphine weekly depot (CAM2038) and hydromorphone blockade in individuals with opioid use disorder: A randomized clinical trial | Ineligible Comparator(s) |
| Wang | 2013 | Can heroin-dependent individuals benefit from a methadone maintenance treatment program before they drop out against medical advice? A 12-month follow-up study | Ineligible Comparator(s) |
| Wang | 2014 | Long-term effects of methadone maintenance treatment with different psychosocial intervention models | Not RCT |
| Wang | 2015 | Comparison of outcomes after 3-month methadone maintenance treatment between heroin users with and without HIV infection: a 3-month follow-up study | Not RCT |
| Wang | 2015 | A randomized, double-blind, controlled study: ji-Tai tablet for the treatment of acute withdrawl syndrome of mild heroin dependence | Ineligible Outcomes |
| Wang | 2018 | Treatment of opioid dependence with buprenorphine/naloxone sublingual tablets: a phase 3 randomized, double-blind, placebo-controlled trial | Included |
| Webster | 2016 | Efficacy and safety of a sublingual buprenorphine/naloxone rapidly dissolving tablet for the treatment of adults with opioid dependence: A randomized trial | Ineligible Comparator(s) |
| Wedam | 2007 | QT-interval effects of methadone, levomethadyl, and buprenorphine in a randomized trial | Ineligible Outcomes |
| Weimand | 2021 | Enablers and hindrances for longer-term abstinence in opioid dependent individuals receiving treatment with extended-release naltrexone: A Norwegian longitudinal recovery trial (NaltRec study) | Not RCT |
| Weinhold | 1992 | Buprenorphine alone and in combination with naloxone in non-dependent humans | Ineligible Population |
| Weiss | 2015 | Long-term outcomes from the National Drug Abuse Treatment Clinical Trials Network Prescription Opioid Addiction Treatment Study | Ineligible Comparator(s) |
| Weiss | 2017 | Pain severity and subsequent opioid use during buprenorphine-naloxone treatment of prescription opioid-dependent patients with chronic pain | Conference Abstract Only |
| White | 2001 | Naltrexone/CBT psychotherapy trial in an opiate-dependent parolee population: initial participant demographic comparison with a local criminal justice population | Conference Abstract Only |
| White | 2009 | Open-label dose-finding trial of buprenorphine implants (Probuphine) for treatment of heroin dependence | Ineligible Comparator(s) |
| Whiteside | 2020 | Emergency department longitudinal integrated care: a pilot randomized clinical trial of a multi-component intervention for patients with opioid use | Conference Abstract Only |
| Williams | 2017 | Long-term follow-up study of community-based patients receiving XR-NTX for opioid use disorders | Not RCT |
| Wilson | 1974 | Low-dosage use of methadone in extended detoxification: an experimental comparison | Ineligible Comparator(s) |
| Wilson | 1993 | Methadone combined with clonidine versus clonidine alone in opiate detoxification | Not RCT |
| Wilson | 2020 | Safety and tolerability of ALO-02 (oxycodone hydrochloride and sequestered naltrexone hydrochloride) extended-release capsules in older patients: a pooled analysis of two clinical trials | Ineligible Population |
| Winklbaur | 2008 | Quality of life in patients receiving opioid maintenance therapy: A comparative study of slow-release morphine versus methadone treatment | Ineligible Outcomes |
| Wittchen | 2005 | Buprenorphine and methadone in the treatment of opioid dependence: Methods and design of the COBRA study | Not RCT |
| Wittchen | 2011 | The role of different substitution drugs and dosage in long-term opiate maintenance treatment | Not RCT |
| Wolstein | 2009 | A randomized, open-label trial comparing methadone and Levo-Alpha- Acetylmethadol (LAAM) in maintenance treatment of opioid addiction | Ineligible Comparator(s) |
| Woody | 1983 | Psychotherapy for opiate addicts. Does it help? | Ineligible Comparator(s) |
| Woody | 2013 | Methadone and suboxone for subutex injectors: Primary outcomes of pilot RCT | Conference Abstract Only |
| Woody | 2021 | Extended release injectable naltrexone before vs after release: A randomized trial of opioid addicted persons who are in prison | Ineligible Comparator(s) |
| Wright | 2007 | Buprenorphine versus dihydrocodeine for opiate detoxification in primary care: A randomised controlled trial | Ineligible Comparator(s) |
| Wright | 2011 | Comparison of methadone and buprenorphine for opiate detoxification (LEEDS trial): a randomised controlled trial | Included |
| Xuyi | 2014 | Phase I study of injectable, depot naltrexone for the relapse prevention treatment of opioid dependence | Ineligible Outcomes |
| Yaghubi | 2017 | Comparing Effectiveness of Mindfulness-Based Relapse Prevention with Treatment as Usual on Impulsivity and Relapse for Methadone-Treated Patients: a Randomized Clinical Trial | Ineligible Comparator(s) |
| Yancovitz | 1991 | A randomized trial of an interim methadone maintenance clinic | Included |
| Yandoli | 2002 | A comparative study of family therapy in the treatment of opiate users in a London drug clinic | Ineligible Comparator(s) |
| Yaroslavtseva | 2014 | The impact of oral and implantable formulations of naltrexone on overall functioning and social adjustment in opioid dependent patients | Conference Abstract Only |
| Zacny | 1997 | Comparing the subjective, psychomotor and physiological effects of intravenous buprenorphine and morphine in healthy volunteers | Ineligible Population |
| Zarghami | 2012 | Tramadol versus methadone for treatment of opiate withdrawal: A double-blind, randomized, clinical trial | Ineligible Comparator(s) |
| Zhu | 2017 | Baseline characteristics and outcomes associated with long-term opioid abstinence after randomization to methadone versus buprenorphine/naloxone in a multi-site trial | Conference Abstract Only |
| Zhu | 2018 | Correlates of Long-Term Opioid Abstinence After Randomization to Methadone Versus Buprenorphine/Naloxone in a Multi-Site Trial | Ineligible Outcomes |
| Ziaaddini | 2018 | Comparison of Buprenorphine and Buprenorphine/naloxone in Detoxification of Opioid-dependent Men | Ineligible Comparator(s) |
| Ziedonis | 2009 | Predictors of outcome for short-term medically supervised opioid withdrawal during a randomized, multicenter trial of buprenorphine-naloxone and clonidine in the NIDA clinical trials network drug and alcohol dependence | Ineligible Comparator(s) |
