# Supplementary material for: Relative effectiveness of medications for opioid-related disorders: A systematic review and network meta-analysis of randomized controlled trials
Source: PLoS One. 2022 Mar 31;17(3):e0266142. doi: 10.1371/journal.pone.0266142 (PMC8970369; doi:10.1371/journal.pone.0266142)
Supplement: S7 Table — 0 This was because the fixed effects model assumes that all studies share the same common effect, but it may not be reasonable to assume that there is one common effect size. On the other hand, the random effects model assumes that the observed estimates of the treatment effect can vary across studies due to systematic differences in the treatment effect as well as random variations due to chance [Riley, R.D., J.P. Higgins, and J.J. Deeks, Interpretation of random effects meta-analyses. BMJ, 2011. 342: p. d549.]. 1 Comparison with the random effects model. 2 Categorization of risk of bias (RoB) was based on whether the overall RoB was ‘Low risk’ or ‘Some or High risk’ using the RoB 2.0 tool. 3 Publication year was stratified by whether the article was published ‘Before 2010’ or ‘On or after 2010’. (DOCX) [file pone.0266142.s008.docx]

**S7 Table. Deviance information criterion (DIC) for fixed effects model versus random effects model^0^ and meta-regression analysis**

| Model | D-bar | pD | DIC | I-squred |
| --- | --- | --- | --- | --- |
| Fixed effects model | 169.40 | 124.88 | 294.28 | 8% |
| Random effects model | 169.51 | 125.07 | 294.58 | 9% |
| Meta-regression risk of bias^1, 2^ | 168.45 | 125.01 | 293.46 | 8% |
| Meta-regression publication year^1, 3^ | 168.84 | 125.82 | 294.66 | 8% |

0 This was because the fixed effects model assumes that all studies share the same common effect, but it may not be reasonable to assume that there is one common effect size. On the other hand, the random effects model assumes that the observed estimates of the treatment effect can vary across studies due to systematic differences in the treatment effect as well as random variations due to chance*.

1 Comparison with the random effects model

2 Categorization of risk of bias (RoB) was based on whether the overall RoB was ‘Low risk’ or ‘Some or High risk’ using the RoB 2.0 tool

3 Publication year was stratified by whether the article was published ‘Before 2010’ or ‘On or after 2010’

* Riley, R.D., J.P. Higgins, and J.J. Deeks, Interpretation of random effects meta-analyses. BMJ, 2011. 342: p. d549.
