# Supplementary material for: Relative effectiveness of medications for opioid-related disorders: A systematic review and network meta-analysis of randomized controlled trials
Source: PLoS One. 2022 Mar 31;17(3):e0266142. doi: 10.1371/journal.pone.0266142 (PMC8970369; doi:10.1371/journal.pone.0266142)
Supplement: S8 Table — (DOCX) [file pone.0266142.s009.docx]

**S8 Table. Average percentage of treatment retention and Surface under the cumulative ranking (SUCRA) score for each treatment in the network**

|  | Methadone | SROM | Buprenorphine | Naltrexone | Control |
| --- | --- | --- | --- | --- | --- |
| Retention % | 77.6 | 64.1 | 54.3 | 41.0 | 30.1 |
| SUCRA | 0.901 | 0.784 | 0.559 | 0.257 | 0.000 |
