# Supplementary figures and images for: Relative effectiveness of medications for opioid-related disorders: A systematic review and network meta-analysis of randomized controlled trials
Source: PLoS One. 2022 Mar 31;17(3):e0266142. doi: 10.1371/journal.pone.0266142 (PMC8970369; doi:10.1371/journal.pone.0266142)

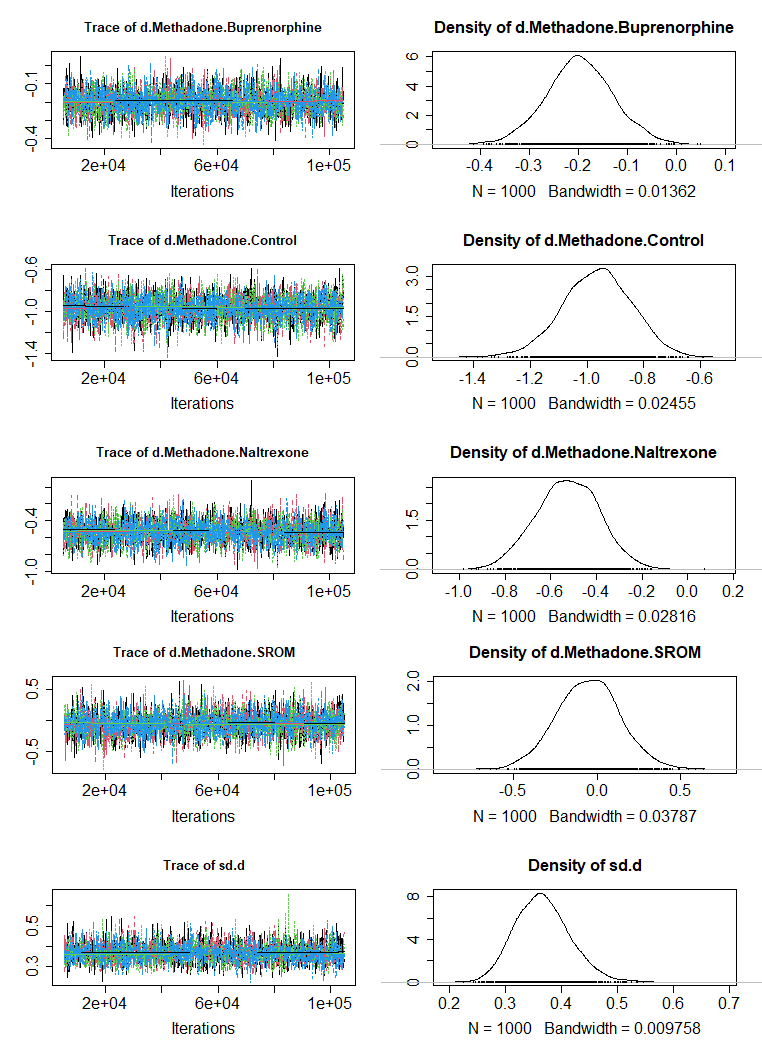

Supplement: S1 Fig — (TIF) [file pone.0266142.s010.tif]

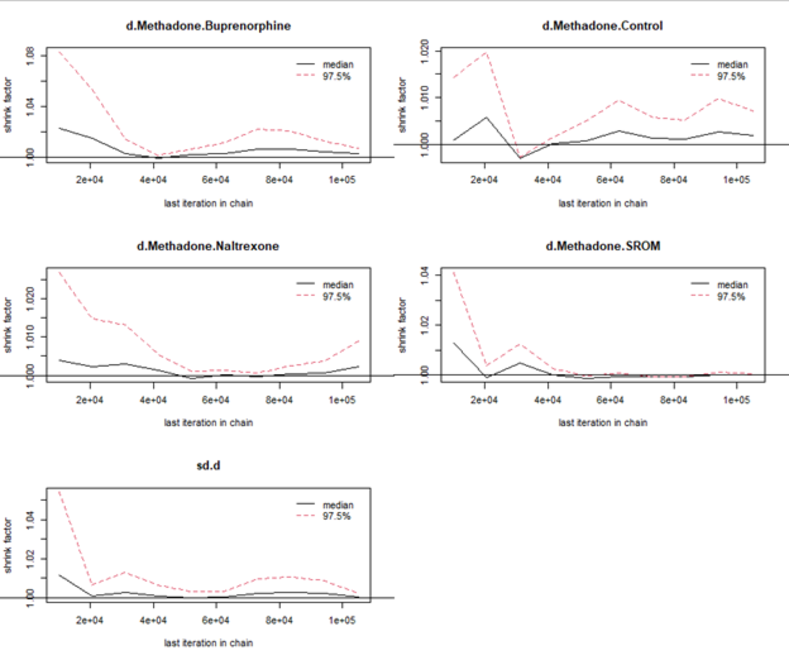

Supplement: S2 Fig — (TIF) [file pone.0266142.s011.tif]

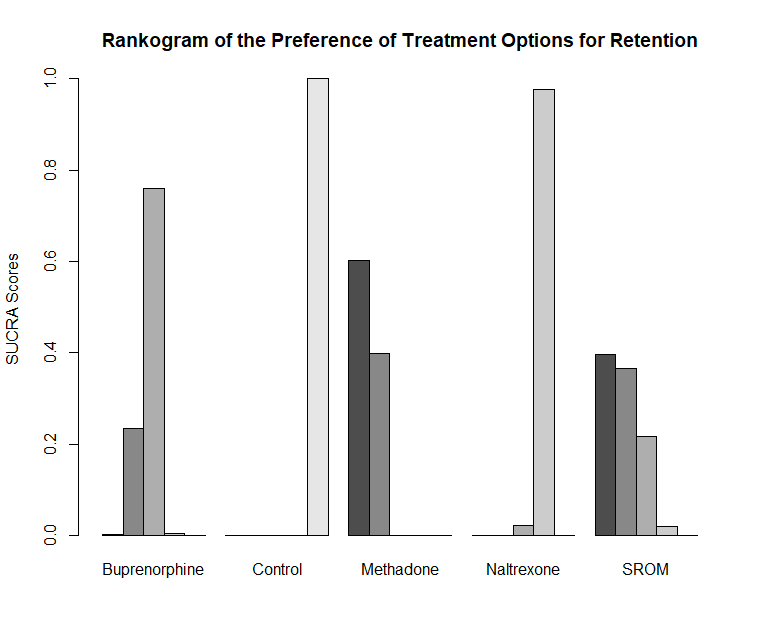

Supplement: S3 Fig — (TIFF) [file pone.0266142.s012.tiff]

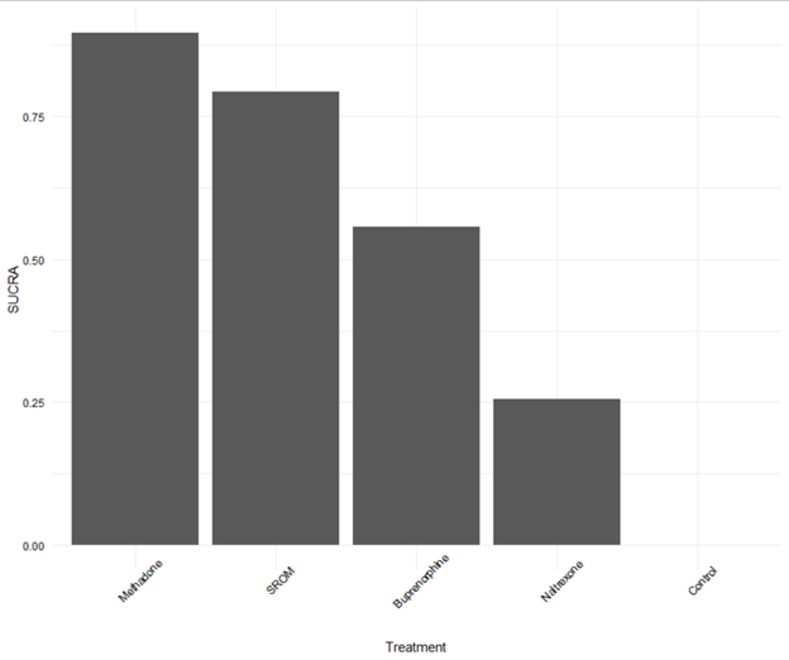

Supplement: S4 Fig — (TIF) [file pone.0266142.s013.tif]

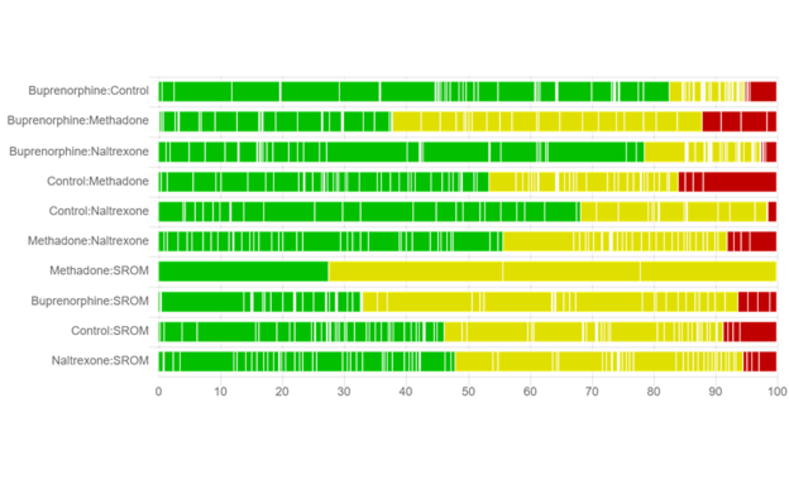

Supplement: S5 Fig — (TIF) [file pone.0266142.s014.tif]

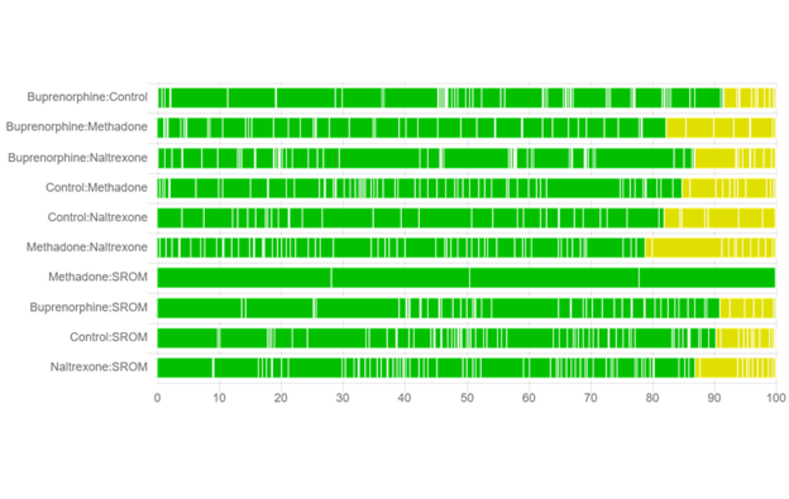

Supplement: S6 Fig — (TIF) [file pone.0266142.s015.tif]
